# Synthesis of chiral *N*-phosphoryl aziridines through enantioselective aziridination of alkenes with phosphoryl azide via Co(II)-based metalloradical catalysis

**DOI:** 10.3762/bjoc.10.129

**Published:** 2014-06-04

**Authors:** Jingran Tao, Li-Mei Jin, X Peter Zhang

**Affiliations:** 1 Department of Chemistry, University of South Florida, Tampa, Florida 33620, USA

**Keywords:** asymmetric aziridination, aziridine, chiral porphyrin, cobalt complex, metalloradical catalysis, organophosphorus, phosphoryl azide

## Abstract

The Co(II) complex of a new *D*_2_-symmetric chiral porphyrin 3,5-DiMes-QingPhyrin, [Co(**P6**)], can catalyze asymmetric aziridination of alkenes with bis(2,2,2-trichloroethyl)phosphoryl azide (TcepN_3_) as a nitrene source. This new Co(II)-based metalloradical aziridination is suitable for different aromatic olefins, producing the corresponding *N*-phosphorylaziridines in good to excellent yields (up to 99%) with moderate to high enantioselectivities (up to 85% ee). In addition to mild reaction conditions and generation of N_2_ as the only byproduct, this new metalloradical catalytic system is highlighted with a practical protocol that operates under neutral and non-oxidative conditions.

## Introduction

Aziridines, the smallest three-membered nitrogen-containing heterocycles, are highly valuable heterocyclic compounds that are widely used in organic synthesis and pharmaceuticals [1–2]. As a result, tremendous efforts have been made for the construction of this class of nitrogen-containing three-membered ring compounds [3–8]. Among synthetic methodologies, catalytic aziridination of alkenes with nitrene sources via “C2 + N1” addition has received the most attention because of the abundance of both alkenes and nitrene sources [9–12]. The enantioselective olefin aziridination is of particular significance due to the streamlined approach for the installation of chiral aziridines, which are versatile intermediates in organic synthesis. To date, several different types of transition metal-based chiral catalysts, such as Mn, Fe, Cu, Rh, Ru and Co complexes, have been demonstrated as effective catalysts in asymmetric olefin aziridination with various nitrene sources, including the widely used iminoiodanes and their in situ variants, chloramine-T, bromamine-T, tosyloxycarbamates and organic azides [9–15]. Among them, the organic azides have recently emerged as attractive alternative nitrene sources for metal-catalyzed aziridination because of many advantages such as ease of preparation, structural diversity, and N_2_ gas as the only byproduct [13–15]. While sulfonyl and aryl azides have been effectively employed for metal-catalyzed asymmetric aziridination [16–19], the catalytic system based on other types of azides, such as phosphoryl azides, remains underdeveloped.

**Scheme 1:**
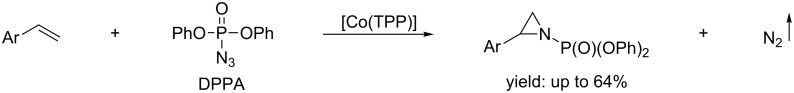
[Co(TPP)]-catalyzed olefin aziridination with DPPA.

**Scheme 2:**
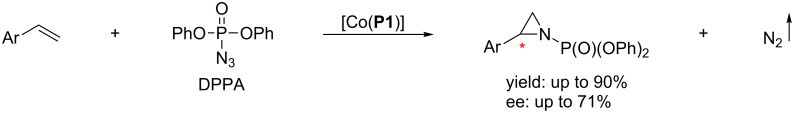
[Co(**P1**)]-catalyzed asymmetric olefin aziridination with DPPA.

Phosphoryl azides, a family of common organic azides that can be directly synthesized from commercially available phosphoryl chlorides, have been recently explored as nitrene sources for transition metal-catalyzed nitrene transfer reactions [20–23]. Their use in a catalytic asymmetric aziridination would provide an attractive approach for the synthesis of valuable chiral phosphorous-containing aziridines, producing nitrogen gas as the only and also environmentally friendly byproduct. Chiral phosphorylated aziridines and their derivatives have been demonstrated with pharmaceutical and other important synthetic applications. In addition to the fundamental and practical significance of the phosphorous-containing aziridines, the easy deprotection of phosphoryl groups makes them even more synthetically useful [24–27]. However very few catalytic systems are available for the direct asymmetric olefin aziridination with phosphoryl azides. In this regard, our group initially reported in 2006 a racemic olefin aziridination system with diphenylphosphoryl azide (DPPA) using Co(II) complexes of common porphyrin ligands as catalysts, including [Co(TPP)] (Scheme 1) [20]. Despite the first demonstration of DPPA as a new nitrene source, this Co(II)-based catalytic transformation, however, suffered from low-yielding formation of the desired aziridine products. To improve the efficiency and control the enantioselectivity of the nitrene transfer process, we then developed a new Co(II)-based metalloradical catalytic system by employing *D*_2_-symmetric chiral amidoporphyrins as the supporting ligands [28]. It was shown that the chiral metalloradical catalyst [Co(**P1**)] (**P1** = 3,5-Di*^t^*Bu-ChenPhyrin) could catalyze the formation of optically enriched phosphoryl aziridine through direct aziridination of alkenes with DPPA (Scheme 2) [21]. While this [Co(**P1**)]/DPPA catalytic system represented the first asymmetric version of olefin aziridination with phosphoryl azide, both the yields and enantioselectivities were moderate even using 10 mol % catalyst loading. It would be desirable if a more effective Co(II)-based metalloradical system could be developed for asymmetric aziridination of alkenes with phosphoryl azides with both improved reactivity and enantioselectivity.

**Figure 1:**
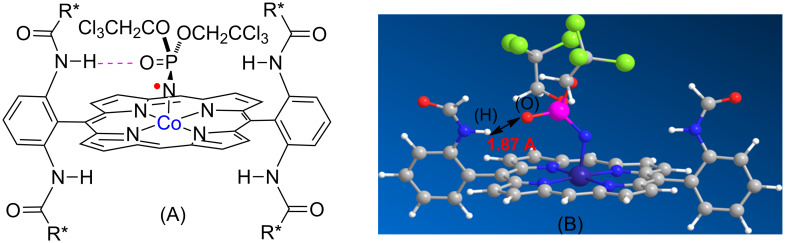
(A) Potential H-bonding interaction in postulated nitrene radical complex of [Co(*D*_2_-Por*)]. R* represents a chiral unit. (B) Geometry corresponding to the minimum energy from simplified computer modeling by molecular mechanics with Spartan 10. The P=O^…^H–N distance of (1.87 Å) suggests possible existence of significant hydrogen bonding interaction. However, besides computer modeling there is no direct experimental evidence for such interactions. For clarity, the other two *meso*-groups of the porphyrin are omitted in both (A) and (B). In addition, the other two amido units below the porphyrin ring are also omitted in (B).

The stable 15e-metalloradicals Co(II) complexes of *D*_2_-symmetric chiral amidoporphyrins ([Co(*D*_2_-Por*)] represent a new type of chiral catalysts that have been demonstrated to be effective for asymmetric olefin aziridination using different types of nitrene sources, particularly with sulfonyl and aryl azides [16,18]. Computational and experimental studies have provided increasing evidences to suggest a stepwise radical mechanism for the Co(II)-catalyzed metalloradical aziridination that involves an unprecedented Co(III)–nitrene radical intermediate [29–34]. It is worthy to note the importance of dual functions of the chiral amide units of the *D*_2_-symmetric chiral amidoporphyrin ligands played in the Co(II)-based metalloradical catalysis (MRC): the rigid amide spacers do not only support and orient the chiral environments toward the cobalt metalloradical center, but also function as potential donors to engage in hydrogen bonding with acceptors located at the nitrene moiety in the Co(III)–nitrene radical intermediate [18,35–36]. These secondary hydrogen bonding interactions are expected to lower the energy barrier of the transition state and thus lead to acceleration of the reaction rate as well as improvement of the stereoselectivity [18,29]. Given that the P=O group can serve as a potential hydrogen bond acceptor, we hypothesized that the resulting Co(III)–nitrene radical intermediate from activation of phosphoryl azides would benefit from a similar hydrogen bonding interaction (Figure 1).

**Figure 2:**
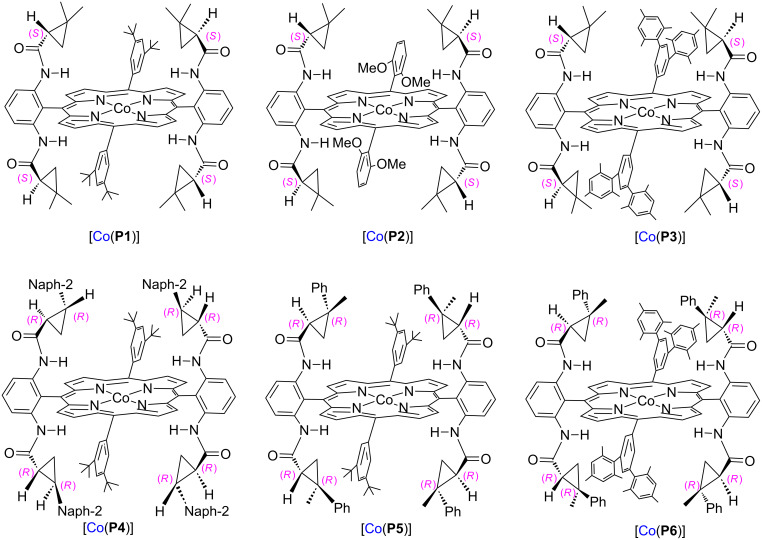
Structures of *D*_2_-symmetric chiral cobalt(II) porphyrins.

With this assumption in mind, we have carried out a systematic study to identify more effective phosphoryl azides and to employ Co(II) complexes of suitable *D*_2_-symmetrical chiral porphyrin ligands ([Co(Por*)]) (Figure 2) for the development of Co(II)-based asymmetric aziridination via MRC to improve the reactivity and selectivity. As the result of this study, herein we wish to report an effective catalytic system for asymmetric olefin aziridination based on the use of bis(2,2,2-trichloroethyl)phosphoryl azide (TcepN_3_) as nitrene source and the employment of new generation of chiral Co(II) metalloradical catalysts. The aziridination via Co(II)-based MRC is applicable for a broad range of aromatic olefins, producing the corresponding *N*-phosphorylated aziridines in good to excellent yields with moderate to high enantioselectivities. In addition to generating N_2_ as the only byproduct, the new metalloradical aziridination process is highlighted by a practical protocol that operates under neutral and non-oxidative reaction conditions without the need of any additives.

## Results and Discussion

Our initial study was focused on the aziridination reaction of styrene (**1a**) as a model reaction and [Co(TPP)] (TPP = 5,10,15,20-tetraphenylporphyrin) as catalyst to search for a more effective phosphoryl azide (Table 1). In the presence of 10 mol % of [Co(TPP)], the phosphoryl azides **2a–c** were found to be ineffective nitrene sources for the catalytic reaction, with no detectable aziridine product but remaining of the starting azides (Table 1, entries 1–3). It should be noted that azide **2c** was previously shown to be a productive nitrene source for the catalytic aziridination reaction only at a high temperature of 80 °C [20]. Afterwards, we were pleased to find that the phosphoryl azide bis(2,2,2-trichloroethyl)phosphoryl azide (TcepN_3_, **2d**) was an effective nitrene source even at low temperature. For instance, at 40 °C, styrene could be aziridinated with the phosphoryl azide TcepN_3_ in low but significant yield when using [Co(TPP)] as the catalyst (Table 1, entry 4). Subsequent experiments showed that Co(II) complexes of *D*_2_-amidoporphyrin ligands (Figure 2) were more effective catalysts to activate TcepN_3_ for the aziridination reaction. For example, under the similar conditions, the reaction catalyzed by [Co(**P1**)] (**P1** = 3,5-Di*^t^*Bu-ChenPhyrin) [28], gave the desired aziridine in 77% yield and 53% ee even using only 2 mol % of catalyst loading (Table 1, entry 5). The dramatic difference observed in the catalytic performance between [Co(**P1**)] and [Co(TPP)] is in accordance with N-H^…^O=P hydrogen bonding which we assume to play an important role in activating the phosphoryl azide (Figure 1). Further studies showed that the Co(II) complex of the more sterically hindered amidoporphyrin ligand [Co(**P2**)] (**P2** = 2,6-DiMeO-ChenPhyrin) gave almost no reaction (Table 1, entry 6), signifying the steric demand of the catalytic process. Gratifyingly, [Co(**P3**)], in which the 3,5-positions of the *meso*-phenyl rings of the porphyrin were installed with mesityl groups, was found extremely effective in catalyzing this olefin aziridination reaction with TcepN_3_, producing the desired aziridine product in almost quantitative yield although with lower enantioselectivity (Table 1, entry 7). Further improvement in enantioselectivity was achieved when [Co(**P4**)] (**P4** = 3,5-Di*^t^*Bu-Xu(2’-Naph)Phyrin), a second-generation MRC catalyst that was previously shown to be optimal for asymmetric olefin aziridination with aryl azides [18], was used as a catalyst, reaching 65% ee but in a poor yield (Table 1, entry 8). To our delight, the use of catalyst [Co(**P5**)] (**P5** = 3,5-Di*^t^*Bu-QingPhyrin), which was shown to be effective in asymmetric intramolecular olefin cyclopropanation [37], led to significant further improvement in enantioselectivity to 81% ee although the yield for the aziridination reaction with TcepN_3_ remained to be low (Table 1, entry 9). These studies on the relationship between catalytic reactivity and the porphyrin ligand structure indicate the importance of both the chiral amido units and the non-chiral substituents of the porphyrin ligand in influencing the catalytic performance of the Co(II) metalloradical center. Accordingly, we designed and synthesized a new *D*_2_-symmetric amidoporphyrin 3,5-DiMes-QingPhyrin (**P6**), whose Co(II) complex [Co(**P6**)] was shown to be the optimal catalyst for this reaction, producing the desired aziridine in 98% yield and 75% ee using only 2 mol % of catalyst loading (Table 1, entry 10). After screening various solvents, it was found that benzene was the solvent of choice for the catalytic process, giving the desired product with high enantioselectivity (81% ee) while maintaining the excellent yield (98%) (Table 1, entries 10–12). Some reduction in reaction temperature (from 40 °C to 35 °C) and time (from 48 h to 36 h) was shown to have no obvious effect on both product yield and enantioselectivity (Table 1, entry 13). However, the catalytic reaction became significantly slower as the temperature further decreased.

**Table 1:** Optimization of catalytic aziridination of styrene with phosphoryl azides by Co(II)-based metalloradical catalysts.^a^

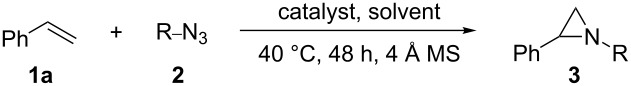					

entry	R–N_3_	catalyst	solvent	yield (%)^b^	ee (%)^c^

1^d^	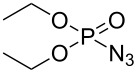 **2a**	[Co(TPP)]	PhCl	0	–
2^d^	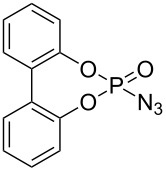 **2b**	[Co(TPP)]	PhCl	0	–
3^d^	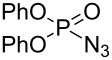 **2c**	[Co(TPP)]	PhCl	0	–
4^d^	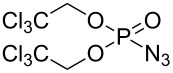 **2d**	[Co(TPP)]	PhCl	11	–
5	**2d**	[Co(**P1**)]	PhCl	77	53
6	**2d**	[Co(**P2**)]	PhCl	<5	nd
7	**2d**	[Co(**P3**)]	PhCl	99	40
8	**2d**	[Co(**P4**)]	PhCl	7	65
9	**2d**	[Co(**P5**)]	PhCl	23	81
10	**2d**	[Co(**P6**)]	PhCl	98	75
11	**2d**	[Co(**P6**)]	PhCF_3_	75	77
12	**2d**	[Co(**P6**)]	C_6_H_6_	98	81
13*^e^*	**2d**	[Co(**P6**)]	C_6_H_6_	99	82

^a^Reaction conditions: 2 mol % of catalyst; olefin:azide = 5:1; [azide] = 0.1 M. ^b^Isolated yield. ^c^Determined by chiral HPLC. ^d^10 mol % of catalyst. ^e^at 35 °C in 36 h.

Under the optimized reaction conditions, we then investigated the scope and limitation of the [Co(**P6**)]/TcepN_3_-based catalytic system for asymmetric olefin aziridination. The Co(II)-catalyzed asymmetric aziridination was shown to be effective for a variety of styrene derivatives with varied electronic and steric properties (Table 2). Similar to styrene, the styrene derivatives with electron-donating groups, such as the *para*-methylated styrene **1b** could be effectively aziridinated to afford the corresponding *N*-phosphoryl aziridine in a high yield with good enantioselectivity (Table 2, entries 1 and 2). In addition to the electron-rich aromatic olefins, styrenes with electron-deficient substituents at various positions were found to be suitable substrates as well for the Co(II)-based asymmetric aziridination. For instance, the *meta-*nitro-substituted styrene **1c** could be aziridinated in a moderate yield and good enantioselectivity (Table 2, entry 3). Interestingly, when the nitro group is located at the *para*-position as in the case of olefin **1d**, the corresponding aziridine was produced in an excellent yield but in low enantioselectivity (Table 2, entry 4). An excellent yield was also achieved for the catalytic aziridination reaction of the sterically hindered substrate *o*-trifluoromethylstyrene (**1e**) (Table 2, entry 5). When *p*-trifluoromethylstyrene (**1f**) was used as the substrate, however, a decrease in reaction yield was observed (Table 2, entry 6). Furthermore, [Co(**P6**)] could effectively catalyze the aziridination reactions of various halogenated styrenes. For example, under similar conditions, *p*-fluorostyrene (**1g**) could be aziridinated with TcepN_3_ in 98% yield with 85% ee (Table 2, entry 7). Like *p*-fluorostyrene, the *p*-chlorostyrene (**1h**) and *p*-bromostyrene (**1i**) were also effective substrates for the metalloradical aziridination system, forming the corresponding chiral aziridines in good yields and enantioselectivities (Table 2, entries 8 and 9). In addition to *p*-bromostyrene, both *m*-bromostyrene (**1j**) and *o*-bromostyrene (**1k**) could also be productively aziridinated (Table 2, entries 10 and 11). Similar to the case of *o*-CF_3_-subtituted styrene **1e** (Table 2, entry 5), the catalytic reaction of the sterically demanding *o*-Br-substituted olefin **1k** gave the desired aziridine in almost quantitative yield as well as high enantioselectivity (Table 2, entry 11). It is worthy to mention that the aryl halide units of these chiral aziridines may be further functionalized via other transformations such as palladium-catalyzed cross-coupling reactions.

**Table 2:** Enantioselective aziridination of olefins with TcepN_3_ catalyzed by [Co(**P6**)].^a^

entry	olefin	aziridine	yield (%)^b^	ee (%)^c^

1	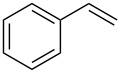 **1a**	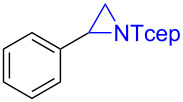 **3a**	99	82
2^d^	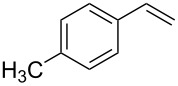 **1b**	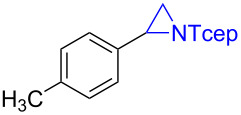 **3b**	86	76
3	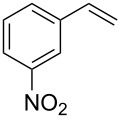 **1c**	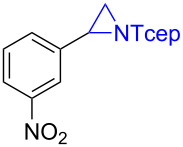 **3c**	66	66
4	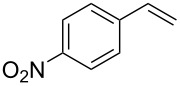 **1d**	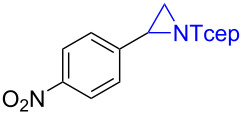 **3d**	90	23
5^e^	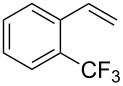 **1e**	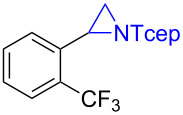 **3e**	98	–
6	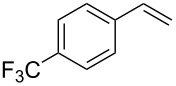 **1f**	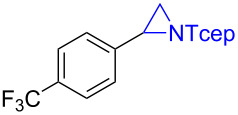 **3f**	64	48
7	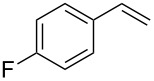 **1g**	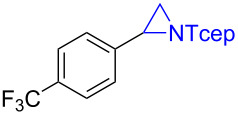 **3g**	98	85
8^d^	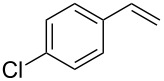 **1h**	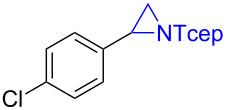 **3h**	74	74
9	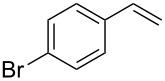 **1i**	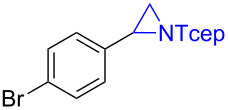 **3i**	98	72
10^d^	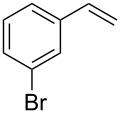 **1j**	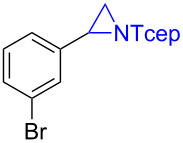 **3j**	85	66
11	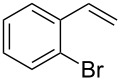 **1k**	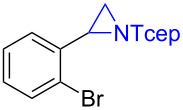 **3k**	99	85

^a^Reaction conditions: 2 mol % of catalyst in the presence of 4 Å MS; olefin:azide = 5:1; [azide] = 0.1 M, benzene as solvent; 35 °C in 36 h. ^b^Isolated yield. ^c^Determined by chiral HPLC. ^d^At 40 °C in 48 h. ^e^The enantiomers could not be resolved.

## Conclusion

In summary, we have shown that the Co(II) complex of the new *D*_2_-symmetric chiral porphyrin 3,5-DiMes-QingPhyrin, [Co(**P6**)], is an effective metalloradical catalyst for asymmetric olefin aziridination with bis(2,2,2-trichloroethyl)phosphoryl azide (TcepN_3_) as a new nitrene source. This [Co(**P6**)]/TcepN_3_-based new aziridination system, which can be operated under neutral and non-oxidative conditions without the need of any additives, is suitable to various aromatic olefins. The resultant enantioenriched *N*-phosphorylaziridines may find potential applications in stereoselective synthesis of both nitrogen- and phosphorous-containing compounds. Efforts are underway to employ phosphoryl azides as effective nitrene sources for other types of organic transformations via Co(II)-based metalloradical catalysis (MRC).

## Supporting Information

File 1 Experimental procedures and characterization data. Copies of ^1^H, ^13^C, and ^31^P NMR spectra and HPLC data for all new compounds.
