# Supplementary material for: Synthesis of chiral N-phosphoryl aziridines through enantioselective aziridination of alkenes with phosphoryl azide via Co(II)-based metalloradical catalysis
Source: Beilstein J Org Chem. 2014 Jun 4;10:1282–9. doi: 10.3762/bjoc.10.129 (PMC4077377; doi:10.3762/bjoc.10.129)
Supplement: File 1 — Experimental procedures and characterization data. Copies of 1H, 13C, and 31P NMR spectra and HPLC data for all new compounds. [file Beilstein_J_Org_Chem-10-1282-s001.pdf]

Supporting Information

for

Synthesis of chiral *N*-phosphoryl aziridines through enantioselective aziridination of alkenes with phosphoryl azide via Co(II)-based metalloradical catalysis

Jingran Tao, Li-Mei Jin and X. Peter Zhang*

Address: Department of Chemistry, University of South Florida, Tampa, Florida 33620

Email: X. Peter Zhang - xpzhang@usf.edu

*Corresponding author

Experimental procedures and characterization data

Copies of ^{1}H, ^{13}C, and ^{31}P NMR spectra and

HPLC data for all new compounds

Table of Contents

Experimental procedures and characterization data S2

References S8

NMR and HPLC spectra of compound **3a–3k**.. S9

General considerations:

Unless otherwise noted, all reactions were carried out under a nitrogen atmosphere in oven-dried glassware following standard Schlenk techniques. Gas-tight syringes were used to transfer liquid reagents and solvents in catalytic reactions. Solvents were freshly distilled/degassed prior to use unless otherwise noted. Thin layer chromatography was performed on Merck TLC plates (silica gel 60 F254). Flash column chromatography was performed with silica gel (60 Å, 230–400 mesh, 32–63 μm). Phosphoryl azides **2a**, **2b**, **2c**, and **2d** were synthesized according to previously reported procedure [1]. Catalysts [Co(**P1**)] [2], [Co(**P2**)] [2], [Co(**P3**)] [3], [Co(**P4**)] [4], [Co(**P5**)] [5] and [Co(**P6**)] [3] were readily prepared according to the literature.

Instrumentation:

Nuclear magnetic resonance (^{1}H NMR and ^{13}C NMR) spectra were recorded on a Varian 400 MHz instrument. Chemical shifts for protons are reported in parts per million downfield from tetramethylsilane and are referenced to the residual protium in the NMR solvent ($CHCl_3$ = 7.24 ppm). Chemical shifts for carbons are reported in parts per million downfield from tetramethylsilane and are referenced to the carbon resonances of the solvent residual peak ($CDCl_3$ = 77.00 ppm). ^{19}F NMR spectra were recorded on a Varian 400 spectrometer (376 MHz), using $CFCl_3$ (δ = 0 ppm) as internal standard. ^{31}P NMR spectra were recorded on a Varian 400 spectrometer (162 MHz), using H_3PO_4 (δ = 0) as external standard. Infrared spectra were measured with a Nicolet Avatar 320 spectrometer with a Smart Miracle accessory. High-resolution mass spectra were obtained on an

Agilent 6220 instrument using electrospray ionization time-of-flight mass spectrometry (ESI-TOF).

General Procedure for catalytic aziridination: A Schlenk tube was filled with 200 mg of 4 Å molecular sieves (MS) that were dried overnight in an oven before use. To the Schlenk tube, the catalyst (2 mol %) and bis(2,2,2-trichloroethyl) phosphoryl azide (0.10 mmol) were added together. The filled tube was capped with a Teflon screw cap, evacuated, backfilled with nitrogen, and then replaced with a rubber septum. After the addition of alkene substrate (0.50 mmol; 5.0 equiv) and the solvent benzene (1.0 mL) via syringe, the Schlenk tube was capped again with the Teflon screw cap and stirred at 35 °C for 36 h. After the completion of the reaction, the desired aziridine product was purified by flash chromatography from the reaction mixture. In most cases, the aziridine product could be visualized on TLC using cerium ammonium molybdate (CAM) or phosphomolybdic acid (PMA) as the stain.

Bis(2,2,2-trichloroethyl)-(2-phenylaziridin-1-yl)phosphonate (3a): ^{1}H NMR (400 MHz, $CDCl_3$): δ 2.37 (ddd, *J* = 15.5, 3.6, 1.2 Hz, 1 H), 2.89 (ddd, *J* = 19.3, 6.1, 1.2 Hz, 1 H), 3.72 (ddd, *J* = 16.6, 6.1, 3.6 Hz, 1 H), 4.58 ~ 4.71 (m, 4 H), 7.29 ~ 7.32 (m, 5 H). ^{13}C NMR (100 MHz, $CDCl_3$): δ 135.87 (d, *J* = 4.8 Hz), 128.59, 128.30, 126.20, 94.84 (dd, *J* = 10.2, 4.2 Hz), 76.87 (dd, *J* = 5.1, 2.2 Hz), 39.02 (d, *J* = 6.1 Hz), 34.90 (d, *J* = 8.2 Hz). ^{31}P NMR (162 MHz, $CDCl_3$): δ 11.96. HPLC analysis: *ee* = 82%. Whelk (98% hexanes: 2% isopropanol, 1.0 mL/min) t_{major} = 24.91 min. t_{minor} = 23.00 min. HRMS (ESI) ($[M+H]^+$) Calcd. for $C_{12}H_{12}Cl_6NO_3P\cdot H^+$: 459.8764, Found: 459.8760.

Bis(2,2,2-trichloroethyl)-(2-(4-methylphenyl)aziridin-1-yl)phosphonate (3b): ^{1}H NMR (400 MHz, $CDCl_3$): δ 2.36 (m, 4 H), 2.87 (dd, J = 19.4, 6.1 Hz, 1 H), 3.69 (ddd, J = 16.6, 6.1, 3.6 Hz, 1 H), 4.73 ~ 4.57 (m, 4 H), 7.14 (d, J = 8.0 Hz, 2 H), 7.19 (d, J = 8.2 Hz, 2 H). ^{13}C NMR (100 MHz, $CDCl_3$): δ 138.12, 132.82 (d, J = 4.9 Hz), 129.267, 126.116, 94.85 (dd, J = 10.4, 4.1 Hz), 76.86 (dd, J = 5.0, 2.9 Hz), 38.99 (d, J = 6.2 Hz), 34.80 (d, J = 8.2 Hz), 21.15. ^{31}P NMR (162 MHz, $CDCl_3$): δ 12.10. HPLC analysis: *ee* = 76%. Whelk (98% hexanes: 2% isopropanol, 1.0 mL/min) t_{major} = 27.13 min, t_{minor} = 24.65 min. HRMS (ESI) ($[M+H]^+$) Calcd. for $C_{13}H_{14}Cl_6NO_3P{\cdot}H^+$: 473.8915, Found: 473.8917.

Bis(2,2,2-trichloroethyl)-(2-(3-nitrophenyl)aziridin-1-yl)phosphonate (3c): ^{1}H NMR (400 MHz, $CDCl_3$): δ 2.39 (dd, J = 15.3, 3.3 Hz, 1 H), 2.97 (dd, J = 18.9, 6.1 Hz, 1 H), 3.81 (ddd, J = 16.4, 6.0, 3.4 Hz, 1 H), 4.85-4.55 (m, 4 H), 7.53 (t, J = 8.2 Hz, 1 H), 7.66 (d, J = 7.6 Hz, 1 H), 8.29 ~ 8.06 (m, 2 H). ^{13}C NMR (100 MHz, $CDCl_3$): δ 148.53, 138.41, 138.36, 132.38, 129.68, 123.30, 121.24, 94.70 (d, J = 10.2 Hz), 76.91 (t, J = 4.9 Hz), 37.96 (d, J = 5.9 Hz), 35.13 (d, J = 8.2 Hz). ^{31}P NMR (162 MHz, $CDCl_3$): δ 11.15. HPLC analysis: *ee* = 66%. Whelk (98% hexanes: 2% isopropanol, 1.0 mL/min) t_{major} = 61.10 min, t_{minor} = 57.34 min. HRMS (ESI) ($[M+H]^+$) Calcd. for $C_{12}H_{11}Cl_6N_2O_5P{\cdot}H^+$: 504.8610, Found: 504.8609.

Bis(2,2,2-trichloroethyl)-(2-(4-nitrophenyl)aziridin-1-yl)phosphonate (3d): ^{1}H NMR (400 MHz, $CDCl_3$): δ 2.37 (dd, J = 15.3, 3.2 Hz, 1 H), 3.21-2.83 (m, 1 H), 3.95 ~ 3.70 (m, 1H), 5.39 ~ 4.12 (m, 4 H), 7.63 ~ 7.44 (m, 2 H), 8.34 ~ 8.11 (m, 2 H). ^{13}C NMR (100 MHz, $CDCl_3$): δ 147.90, 143.410, 127.09, 123.90, 76.92 (t, J = 4.7 Hz), 38.02 (d, J = 5.7 Hz), 35.36 (d, J = 8.2 Hz). ^{31}P NMR (162 MHz, $CDCl_3$): δ 11.16. HPLC analysis: *ee* = 23%. Whelk (98% hexanes: 2% isopropanol, 1.0

mL/min) t_{major} = 54.72 min, t_{minor} = 51.47 min. HRMS (ESI) ($[M+H]^+$) Calcd. for $C_{12}H_{11}Cl_6N_2O_5P{\cdot}H^+$: 504.8610, Found: 504.8620.

Bis(2,2,2-trichloroethyl)-(2-(2-(trifluoromethyl)phenyl)aziridin-1-yl)phosphonate (3e): ^{1}H NMR (400 MHz, $CDCl_3$): δ 2.28 (dd, *J* = 15.3, 3.5 Hz, 1 H), 2.95 (dd, *J* = 18.7, 6.4 Hz, 1 H), 4.14 ~ 3.99 (m, 1 H), 4.84 ~ 4.70 (m, 4 H), 7.42 (t, *J* = 7.5 Hz, 1 H), 7.72 ~ 7.51 (m, 3 H). ^{13}C NMR (100 MHz, $CDCl_3$): δ 134.55, 132.25, 128.06, 127.18, 125.73, 125.67, 94.82, 77,18, 76.88 (t, *J* = 4.9 Hz), 36.26 (d, *J* = 5.5 Hz), 35.00 (d, *J* = 7.4 Hz). ^{31}P NMR (162 MHz, $CDCl_3$): δ 11.85. ^{19}F NMR (376 MHz, $CDCl_3$): δ -59.91. HRMS (ESI) ($[M+H]^+$) Calcd. for $C_{13}H_{11}Cl_6F_3NO_3P{\cdot}H^+$: 527.8633, Found: 527.8657.

Bis(2,2,2-trichloroethyl)-(2-(4-(trifluoromethyl)phenyl)aziridin-1-yl)phosphonate (3f): ^{1}H NMR (400 MHz, $CDCl_3$): δ 2.36 (ddd, *J* = 15.4, 3.4, 1.1 Hz, 1 H), 2.95 (ddd, *J* = 19.0, 6.2, 1.1 Hz, 1 H), 3.77 (ddd, *J* = 16.4, 6.1, 3.5 Hz, 1 H), 5.04 ~ 4.33 (m, 4 H), 7.44 (d, *J* = 8.2 Hz, 2 H), 7.61 (d, *J* = 8.2 Hz, 2 H). ^{13}C NMR (100 MHz, $CDCl_3$): δ 140.09, 130.72, 126.57, 125.60 (d, *J* = 3.7 Hz), 94.75 (d, *J* = 10.1 Hz), 77.186, 76.890 (t, *J* = 4.7 Hz), 38.32(d, *J* = 5.9 Hz), 35.10(d, *J* = 8.1 Hz). ^{31}P NMR (162 MHz, $CDCl_3$): δ 11.51. ^{19}F NMR (376 MHz, $CDCl_3$): δ -62.66. HPLC analysis: *ee* = 48%. Whelk (98% hexanes: 2% isopropanol, 0.5 mL/min) t_{major} = 40.40 min, t_{minor} = 37.39 min. HRMS (ESI) ($[M+H]^+$) Calcd. for $C_{13}H_{11}Cl_6F_3NO_3P{\cdot}H^+$: 527.8633, Found: 527.8643.

Bis(2,2,2-trichloroethyl)-(2-(4-fluorophenyl)aziridin-1-yl)phosphonate (3g): ^{1}H NMR (400 MHz, $CDCl_3$): δ 2.33 (ddd, *J* = 15.4, 3.5, 1.1 Hz, 1 H), 2.88 (ddd, *J* = 19.2, 6.1, 1.1 Hz, 1 H), 3.70 (ddd, *J* = 16.6, 6.1, 3.5 Hz, 1 H), 4.65 (m, 4 H), 7.09-6.94 (m, 2 H), 7.27 (m, 2 H). ^{13}C NMR (100 MHz, $CDCl_3$): δ 163.91, 161.45,

131.68, 127.91 (d, J = 8.1 Hz), 115.61 (d, J = 21.6 Hz), 94.82 (d, J = 7.7 Hz), 76.89, 38.43 (d, J = 5.9 Hz), 34.94 (d, J = 8.1 Hz). ^{31}P NMR (162 MHz, $CDCl_3$): δ 11.81. ^{19}F NMR (376 MHz, $CDCl_3$): δ -113.52 (m). HPLC analysis: *ee* = 85%. Whelk (98% hexanes: 2% isopropanol, 1.0 mL/min) t_{major} = 24.28 min, t_{minor} = 22.31 min. HRMS (ESI) ($[M+H]^+$) Calcd. for $C_{12}H_{11}Cl_6FNO_3P \cdot H^+$: 477.8665, Found: 477.8667.

Bis(2,2,2-trichloroethyl)-(2-(4-chlorophenyl)aziridin-1-yl)phosphonate (3h): ^{1}H NMR (400 MHz, $CDCl_3$): δ 2.33 (dd, J = 15.4, 3.5 Hz, 1 H), 2.90 (dd, J = 19.2, 6.1 Hz, 1 H), 3.69 (ddd, J = 16.5, 6.1, 3.5 Hz, 1 H), 4.74 ~ 4.57 (m, 4 H), 7.23 (d, J = 8.8 Hz, 2 H), 7.30 (d, J = 12 Hz, 2 H). ^{13}C NMR (100 MHz, $CDCl_3$): δ 134.48 (d, J = 5.1 Hz), 134.14, 128.81, 127.54, 94.77 (dd, J = 10.1, 2.7 Hz), 76.88 (t, J = 4.0 Hz), 38.36 (d, J = 6.1 Hz), 34.97 (d, J = 8.2 Hz). ^{31}P NMR (162 MHz, $CDCl_3$): δ 11.71. HPLC analysis: *ee* = 74%. Whelk (98% hexanes: 2% isopropanol, 1.0 mL/min) t_{major} = 24.26 min, t_{minor} = 22.11 min. HRMS (ESI) ($[M+H]^+$) Calcd. for $C_{12}H_{11}Cl_7NO_3P \cdot H^+$: 493.8369, Found: 493.8374.

Bis(2,2,2-trichloroethyl)-(2-(4-bromophenyl)aziridin-1-yl)phosphonate (3i): ^{1}H NMR (400 MHz, $CDCl_3$): δ 2.32 (ddd, J = 15.4, 3.5, 1.1 Hz, 1 H), 2.89 (ddd, J = 19.2, 6.1, 1.1 Hz, 1 H), 3.67 (ddd, J = 16.5, 6.1, 3.5 Hz, 1 H), 4.77 ~ 4.55 (m, 4 H), 7.18 (m, 2 H), 7.50 ~ 7.42 (m, 2 H). ^{13}C NMR (100 MHz, $CDCl_3$): δ 135.03 (d, J = 5.0 Hz), 131.75, 127.86, 122.24, 94.77 (dd, J = 10.2, 3.0 Hz), 76.87 (t, J = 4.8 Hz), 38.42 (d, J = 6.0 Hz), 34.94 (d, J = 8.1 Hz). ^{31}P NMR (162 MHz, $CDCl_3$): δ 11.68. HPLC analysis: *ee* = 72%. Whelk (98% hexanes: 2% isopropanol, 1.0 mL/min) t_{major} = 27.48 min, t_{minor} = 24.80 min. HRMS (ESI) ($[M+H]^+$) Calcd for $C_{12}H_{11}BrCl_6NO_3P \cdot H^+$: 537.7864, Found: 537.7860.

Bis(2,2,2-trichloroethyl)-(2-(3-bromophenyl)aziridin-1-yl)phosphonate (3j): ^{1}H NMR (400 MHz, $CDCl_3$): δ 2.34 (ddd, *J* = 15.4, 3.4, 1.1 Hz, 1 H), 2.90 (ddd, *J* = 19.1, 6.13, 1.1 Hz, 1 H), 3.68 (ddd, *J* = 16.5, 6.1, 3.5 Hz, 1 H), 4.80 ~ 4.57 (m, 4 H), 7.45 (ddd, *J* = 7.5, 3.3, 1.8 Hz, 2 H), 7.24 (m, 2 H). ^{13}C NMR (100 MHz, $CDCl_3$): δ 138.32 (d, *J* = 5.2 Hz), 131.43, 130.13, 129.16, 125.02, 122.74, 94.74 (dd, *J* = 10.2, 2.3 Hz), 76.88 (t, *J* = 4.4 Hz), 38.22(d, *J* = 6.0 Hz), 34.93 (d, *J* = 8.2 Hz). ^{31}P NMR (162 MHz, $CDCl_3$): δ 11.55. HPLC analysis: *ee* = 66%. Whelk (98% hexanes: 2% isopropanol, 1.0 mL/min) t_{major} = 26.83 min, t_{minor} = 23.72 min. HRMS (ESI) ($[M+H]^+$) Calcd for $C_{12}H_{11}BrCl_6NO_3P{\cdot}H^+$: 537.7864, Found: 537.7866.

Bis(2,2,2-trichloroethyl)-(2-(2-bromophenyl)aziridin-1-yl)phosphonate (3k): ^{1}H NMR (400 MHz, $CDCl_3$): δ 2.25 (ddd, *J* = 15.2, 3.6, 1.3 Hz, 1 H), 2.94 (ddd, *J* = 18.7, 6.2, 1.3 Hz, 1 H), 4.00 (ddd, *J* = 16.4, 6.2, 3.5 Hz, 1 H), 4.79 ~ 4.61 (m, 4 H), 7.21 ~ 7.13 (m, 1 H), 7.38 ~ 7.26 (m, 2 H), 7.54 (dd, *J* = 8.0, 1.1 Hz, 1 H). ^{13}C NMR (100 MHz, $CDCl_3$): δ 135.44 (d, *J* = 5.6 Hz), 132.44, 129.55, 127.65, 127.46, 123.35, 94.82 (d, *J* = 10.1 Hz), 76.91 (t, *J* = 6.3 Hz), 39.32 (d, *J* = 5.6 Hz), 34.54 (d, *J* = 7.9 Hz). ^{31}P NMR (162 MHz, $CDCl_3$): δ 12.00. HPLC analysis: *ee* = 85%. Whelk (98% hexanes: 2% isopropanol, 1.0 mL/min) t_{major} = 23.95 min, t_{minor} = 22.51 min. HRMS (ESI) ($[M+H]^+$) Calcd for $C_{12}H_{11}BrCl_6NO_3P{\cdot}H^+$: 537.7864, Found: 537.7864.

References

1. Lu, H. J.; Tao, J. R.; Jones, J. E.; Wojtas, L.; Zhang, X. P. *Org. Lett.* **2010,** *12,* 1248–1251. doi:10.1021/ol100110z
2. Chen, Y.; Fields, K. B.; Zhang, X. P. *J. Am. Chem. Soc.* **2004,** *126,* 14718–14719. doi:10.1021/ja044889l
3. Cui, X.; Xu, X.; Lu, H.; Zhu, S.; Wojtas, L.; Zhang, X. P. *J. Am. Chem. Soc.* **2011,** *133,* 3304–3307. doi:10.1021/ja111334j
4. Jin, L.-M.; Xu, X.; Lu, H.; Cui, X.; Wojtas, L.; Zhang, X. P. *Angew. Chem., Int. Ed.* **2013,** *52,* 5309–5313. doi:10.1002/anie.201209599
5. Xu, X.; Lu, H.; Ruppel, J. V.; Cui, X.; Lopez de Mesa, S.; Wojtas, L.; Zhang, X. P. *J. Am. Chem. Soc.* **2011,** *133,* 15292–15295. doi:10.1021/ja2062506

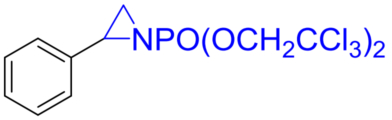

3a

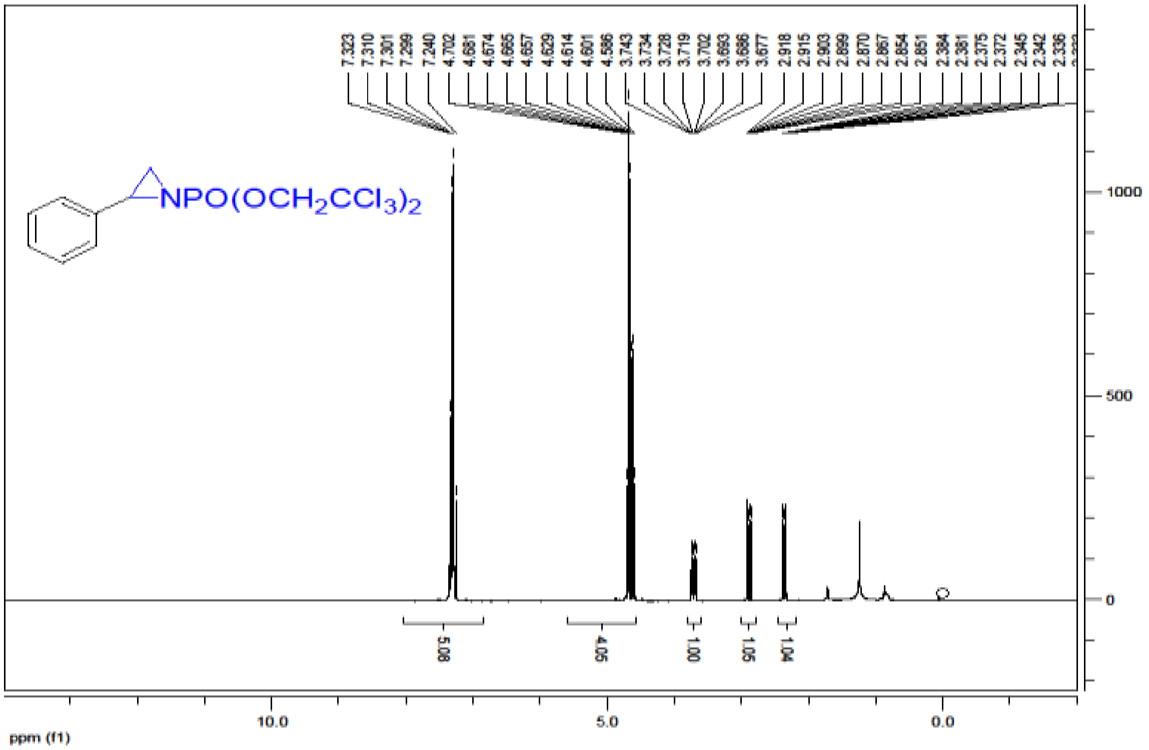

Figure S1. ^{1}H NMR of compound **3a**.

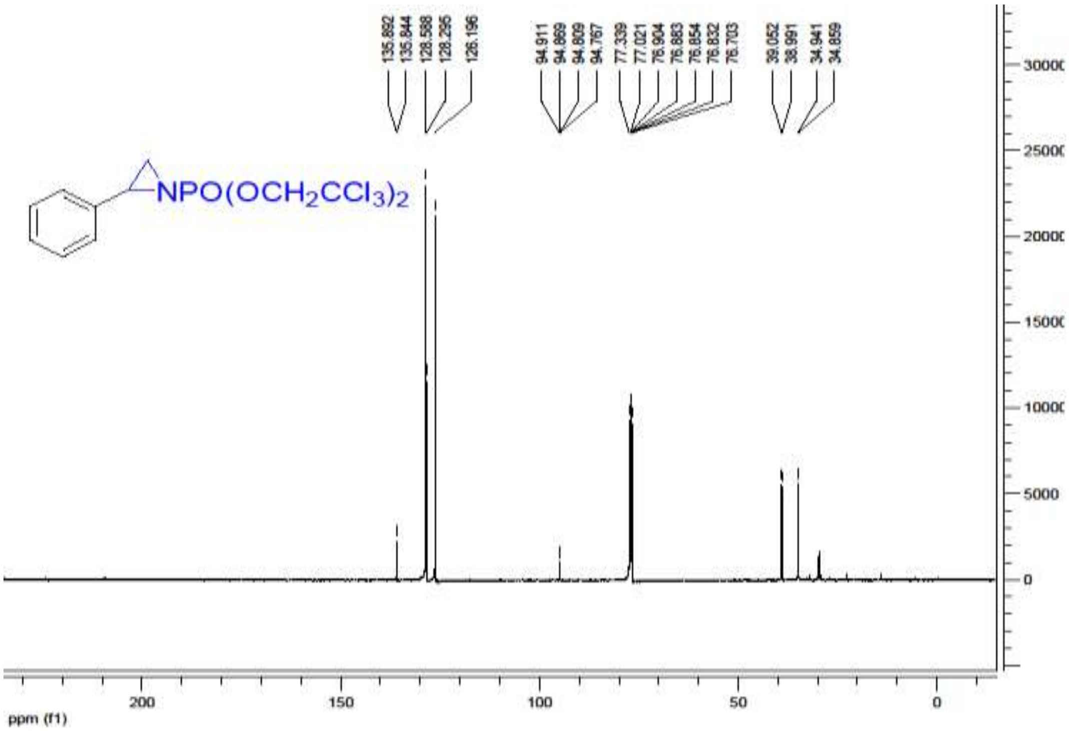

Figure S2. ^{13}C NMR of compound **3a**.

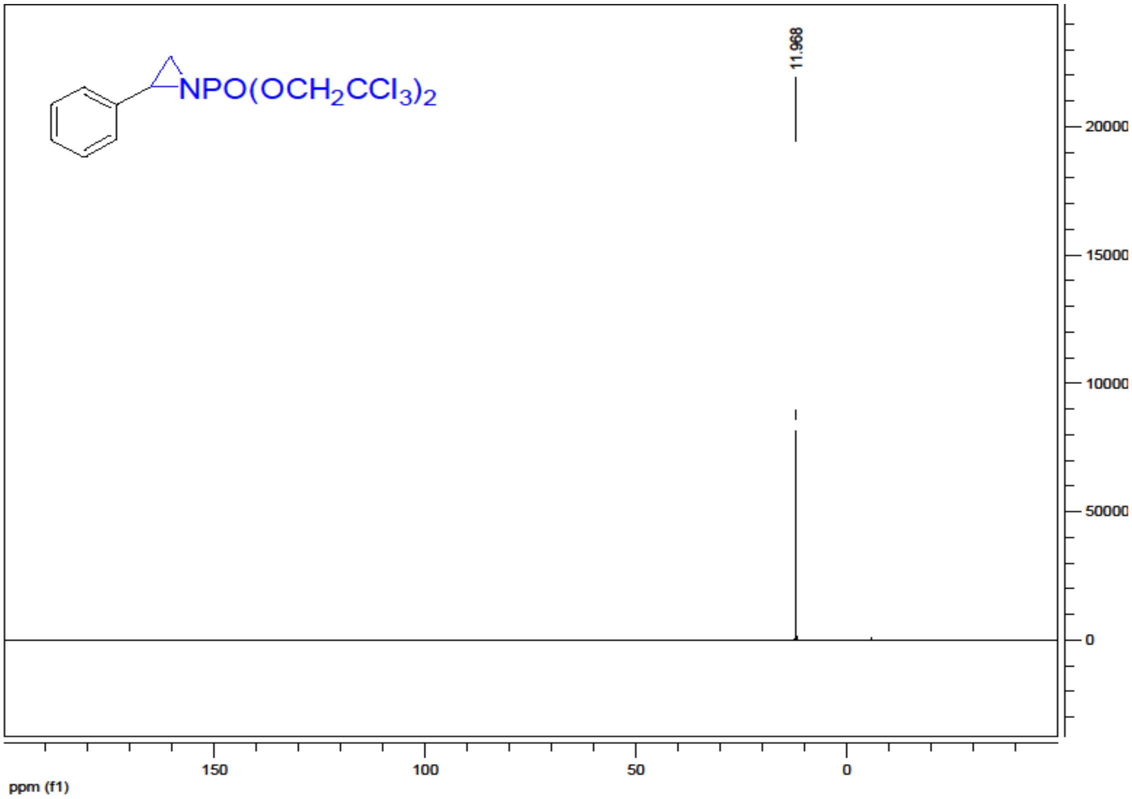

Figure S3. ^{31}P NMR of compound **3a**.

JRT-VI-84-WK2%1ml60min
C:\EZStart\Projects\Default\Method\shifatest_2,5-dimehoxy.met
C:\EZStart\Projects\Default\Data\JRT-VI-84-WK2%1ml60min

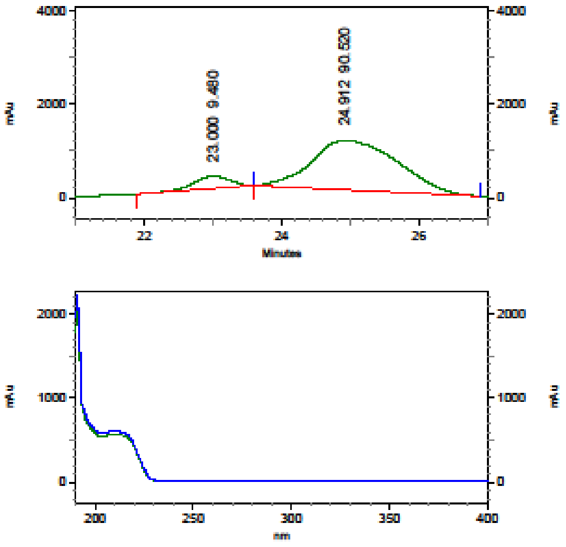

3: 209 nm, 4 nm
Results

Pk # Name	Retention Time	Area Percent
1	23.000	9.480
2	24.912	90.520
Totals		100.000

Figure S4. HPLC of compound **3a**.

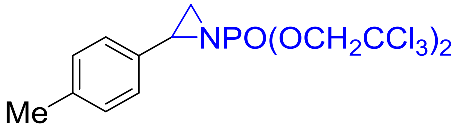

3b

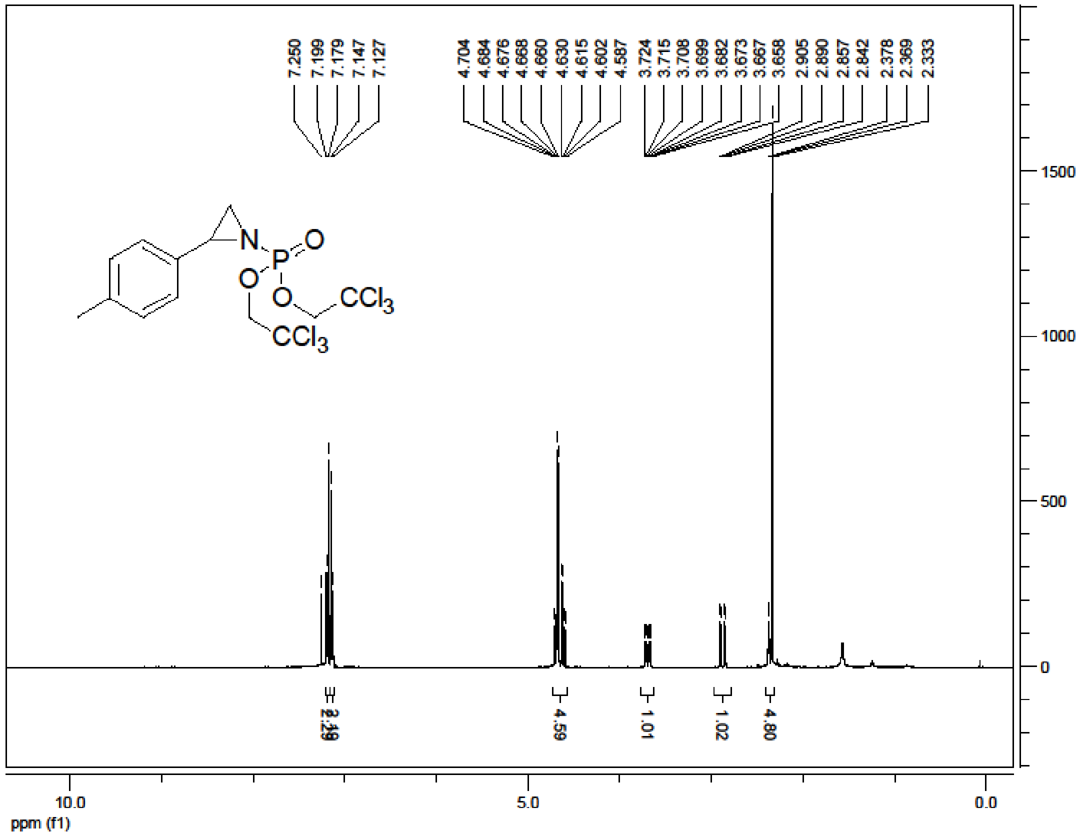

Figure S5. ^{1}H NMR of compound **3b**.

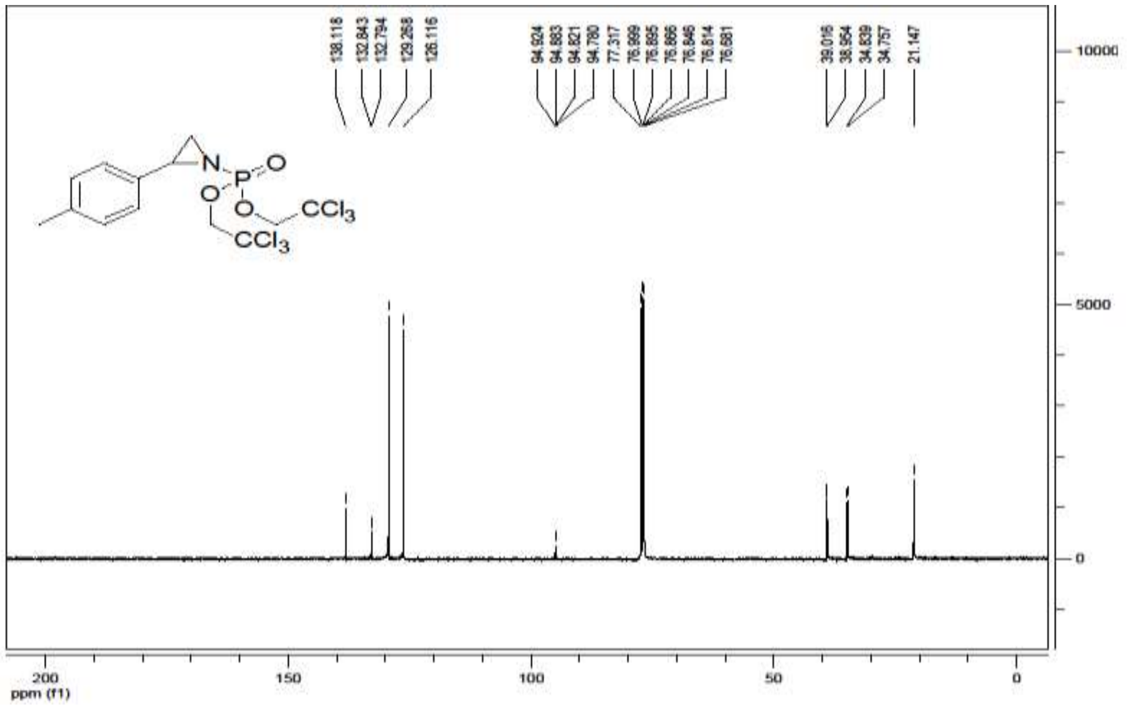

Figure S6. ^{13}C NMR of compound **3b**.

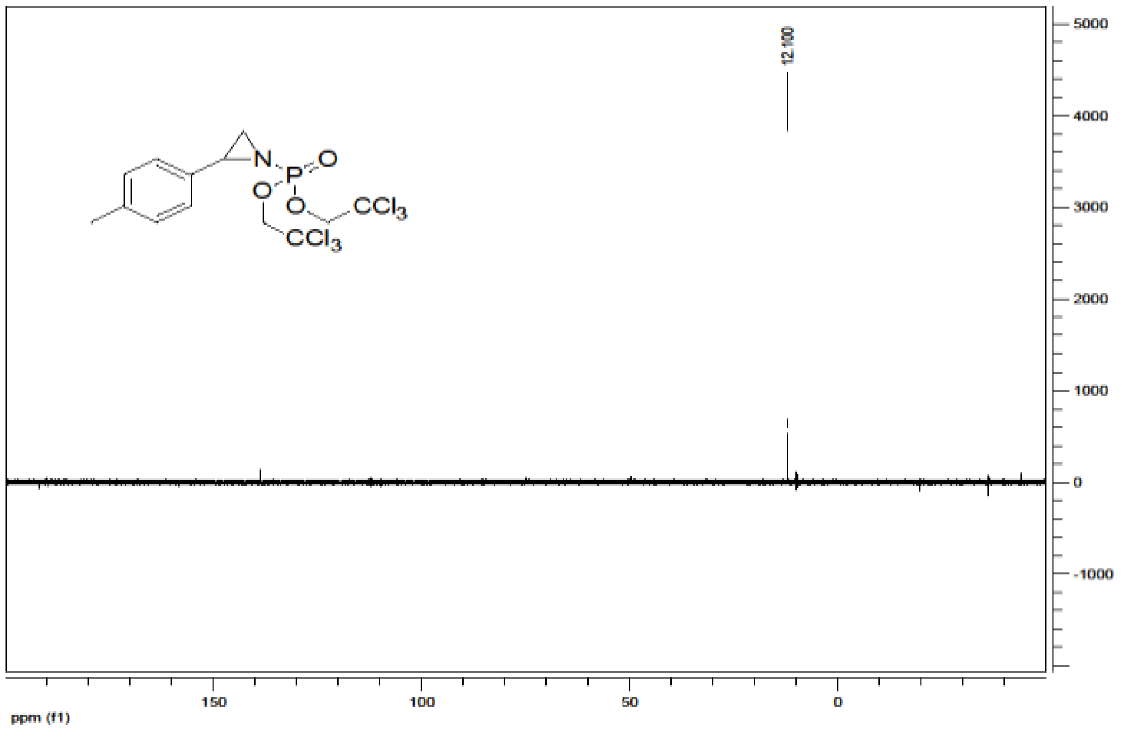

Figure S7. ^{31}P NMR of compound **3b**.

JRT-VI-1423-WK2%1ML
C:\EZStart\Projects\Default\Method\shifatest_2,5-dimehoxy.met
C:\EZStart\Projects\Default\Data\JRT-VI-1423-WK2%1ML

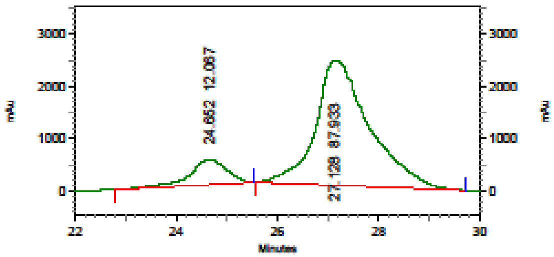

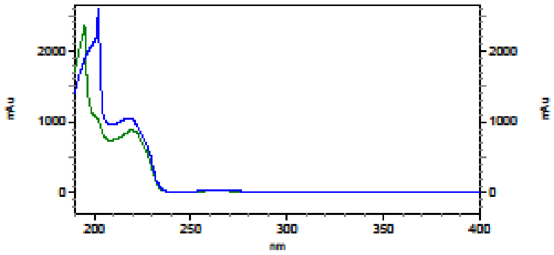

5: 207 nm, 4 nm
Results

Pk #	Name	Retention Time	Area Percent
1		24.652	12.067
2		27.128	87.933
Totals			100.000

Figure S8. HPLC of compound **3b**.

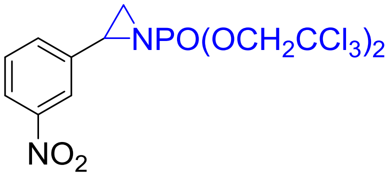

3c

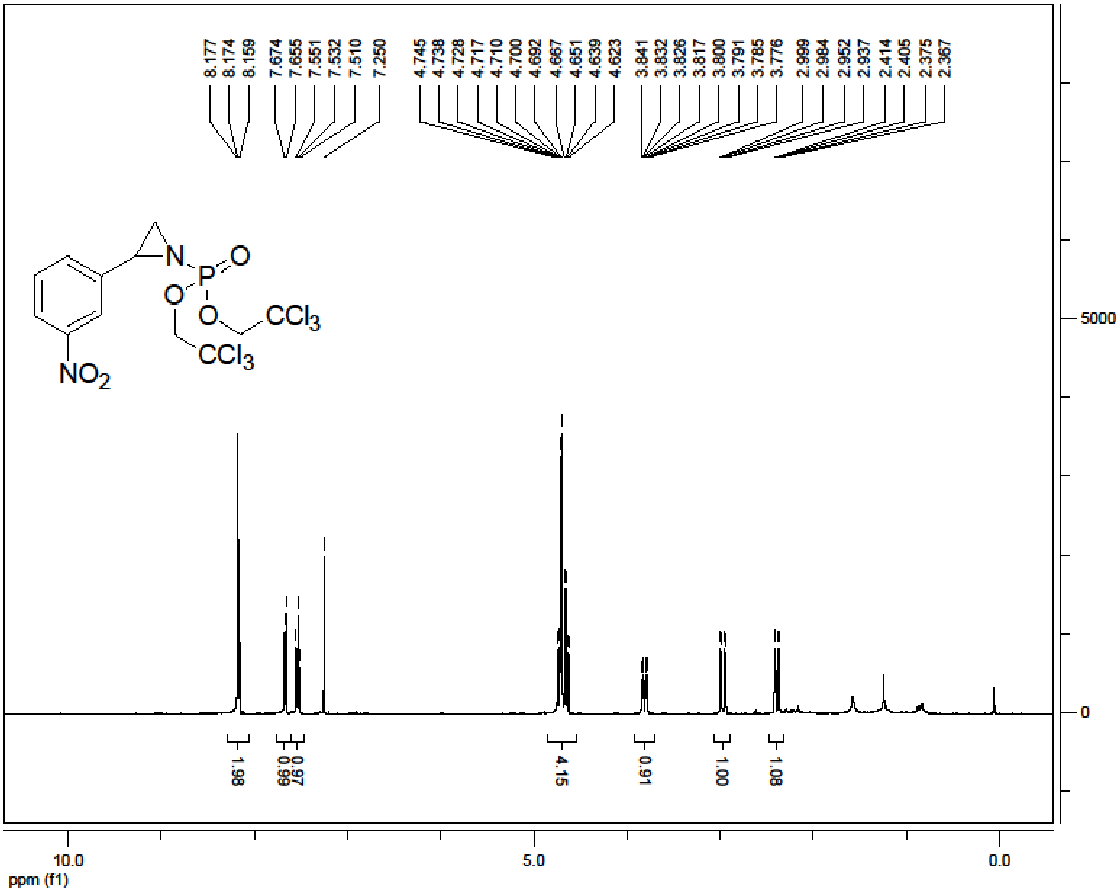

Figure S9. ^{1}H NMR of compound **3c**.

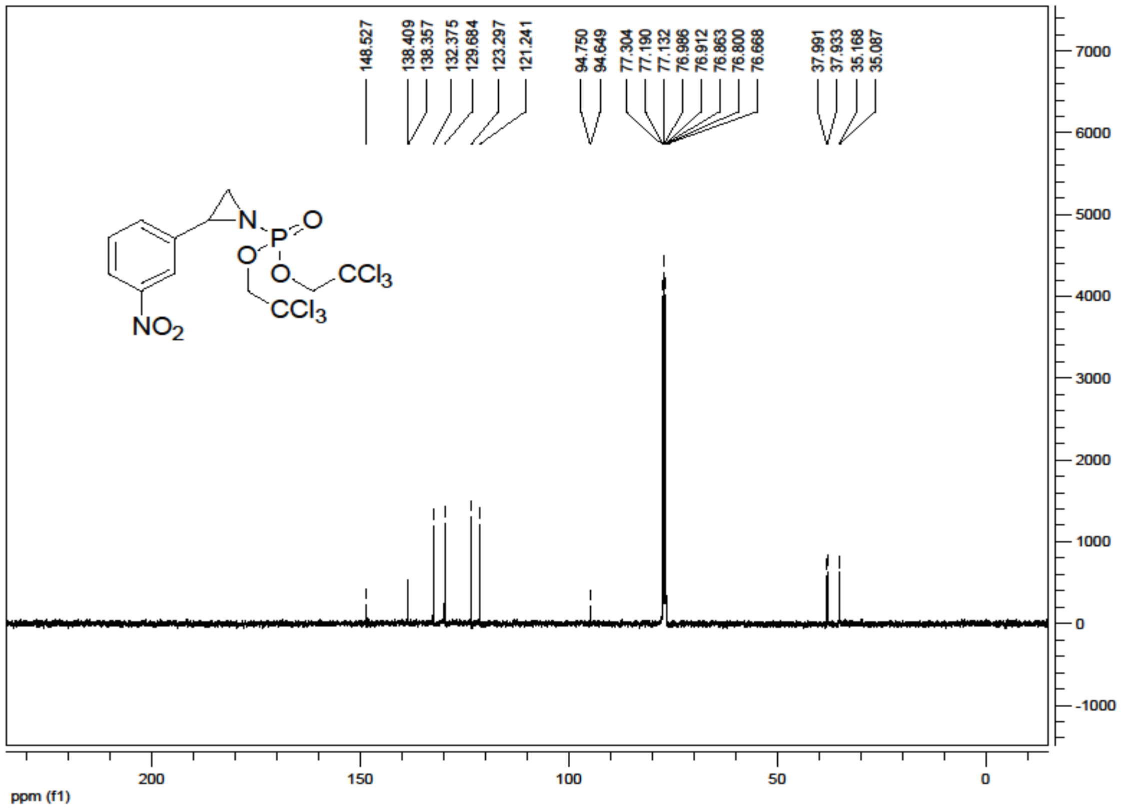

Figure S10. ^{13}C NMR of compound **3c**.

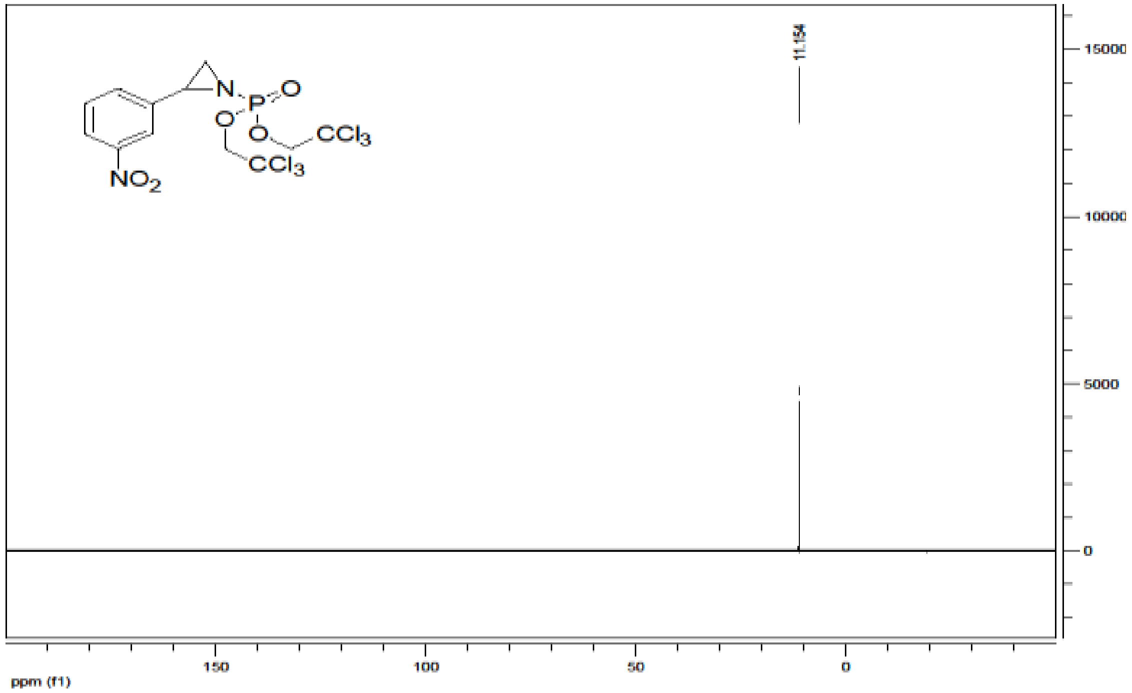

Figure S11. ^{31}P NMR of compound **3c**.

JRT-VI-219A-WK2%1ML150MIN
C:\EZStart\Projects\Default\Method\shifatest_2,5-dimehoxy.met
C:\EZStart\Projects\Default\Data\JRT-VI-219A-WK2%1ML150MIN

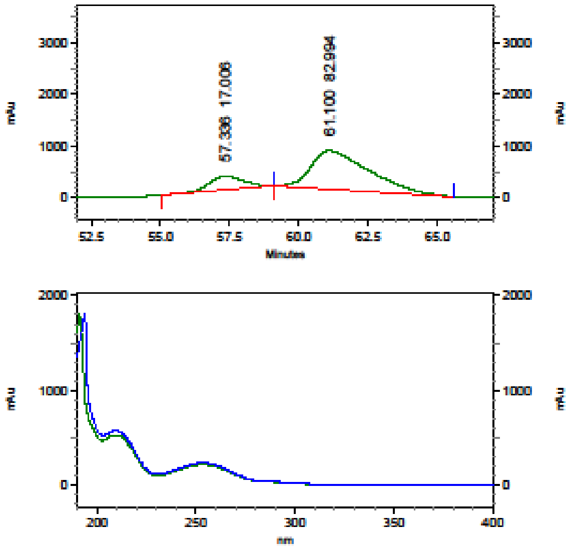

5: 207 nm, 4 nm
Results

Pk #	Name	Retention Time	Area Percent
1		57.336	17.006
2		61.100	82.994
Totals			100.000

Figure S12. HPLC of compound **3c**.

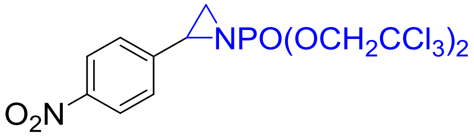

3d

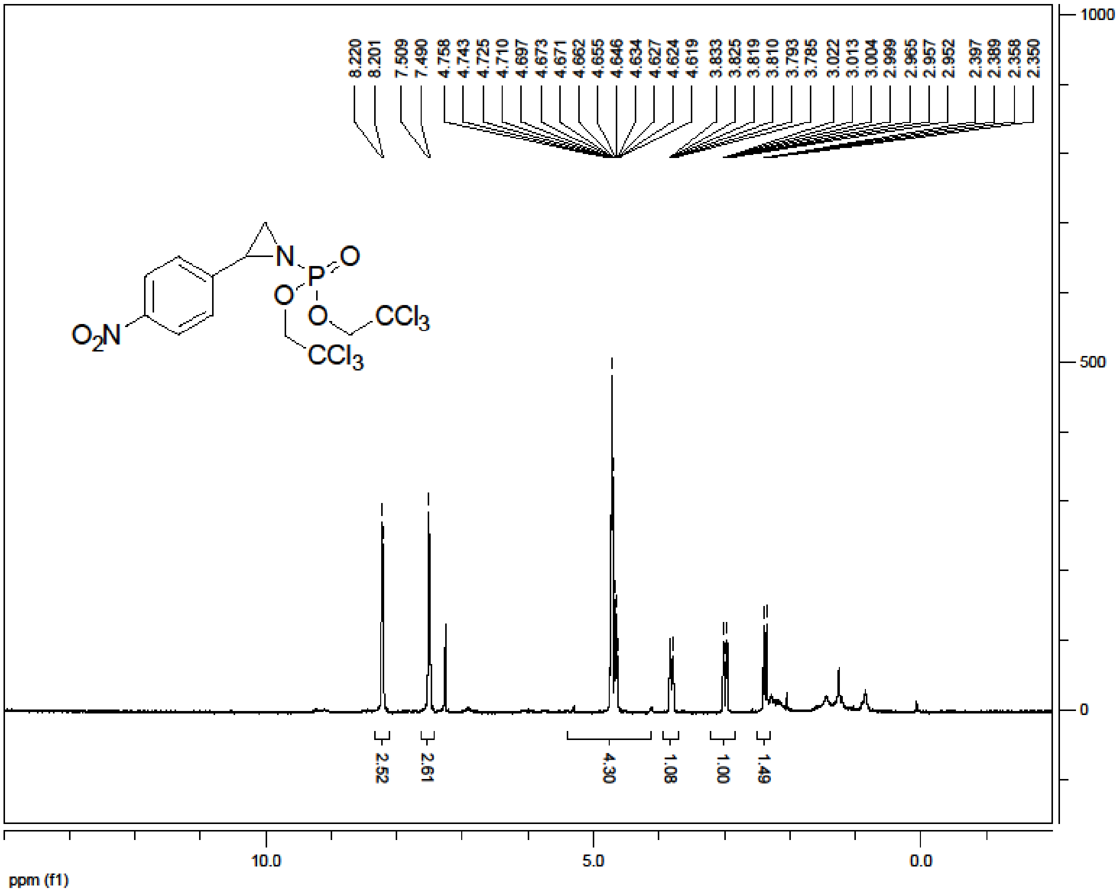

Figure S13. ^{1}H NMR of compound **3d**.

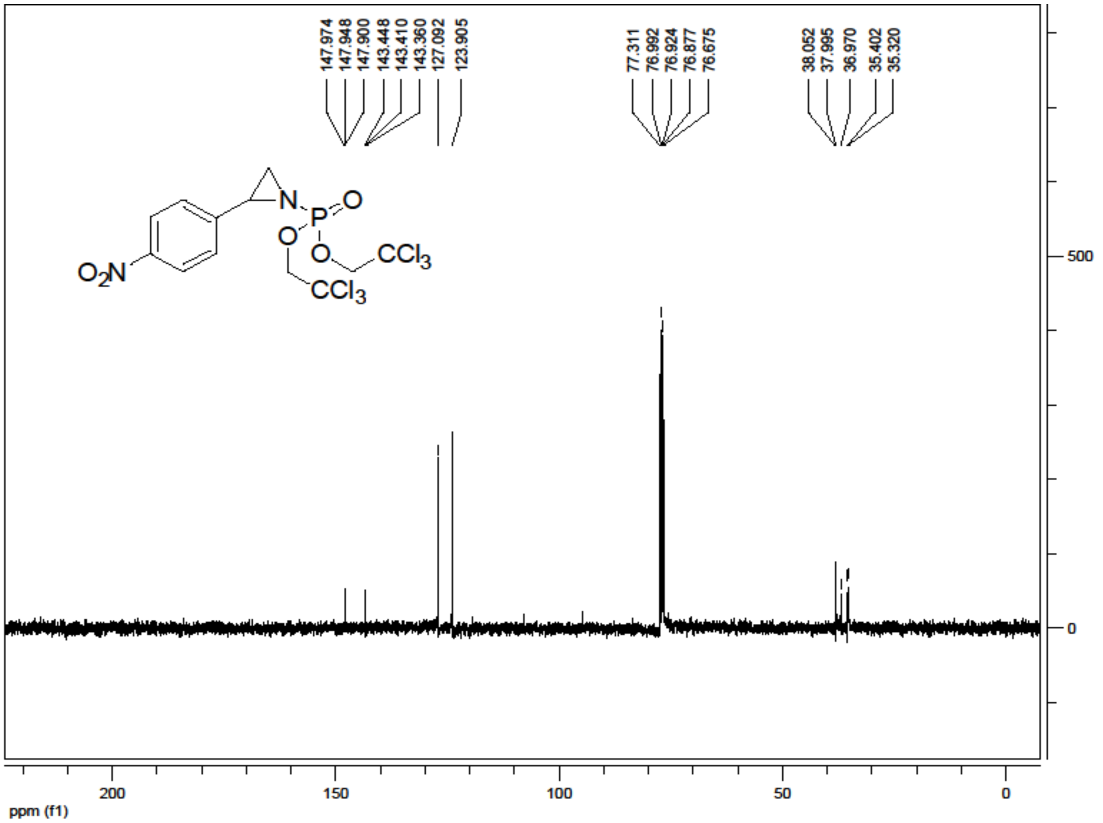

Figure S14. ^{13}C NMR of compound **3d**.

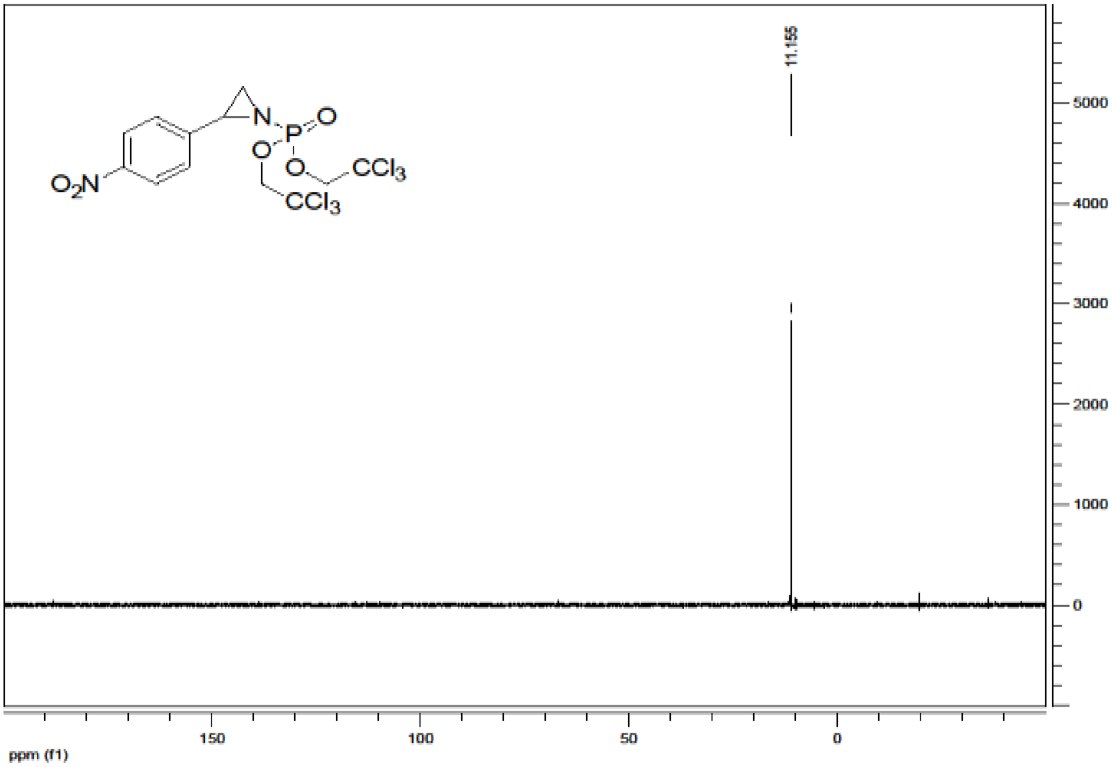

Figure S15. ^{31}C NMR of compound **3d**.

JRT-VI-215-WK2%1ML150MIN
C:\EZStart\Projects\Default\Method\shifatest_2,5-dimehoxy.met
C:\EZStart\Projects\Default\Data\JRT-VI-215-WK2%1ML150MIN

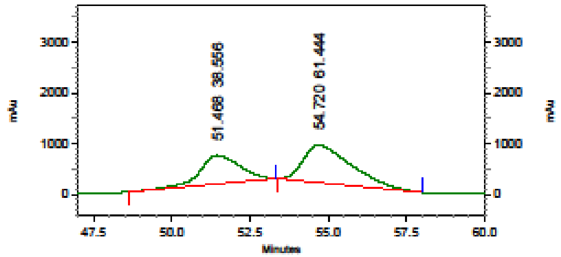

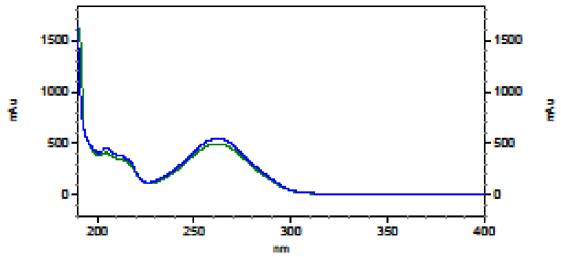

5: 207 nm, 4 nm
Results

Pk #	Name	Retention Time	Area Percent
1		51.468	38.556
2		54.720	61.444
Totals			100.000

Figure S16. HPLC of compound **3d**.

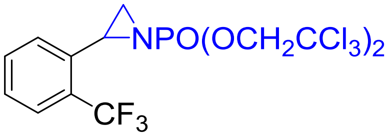

3e

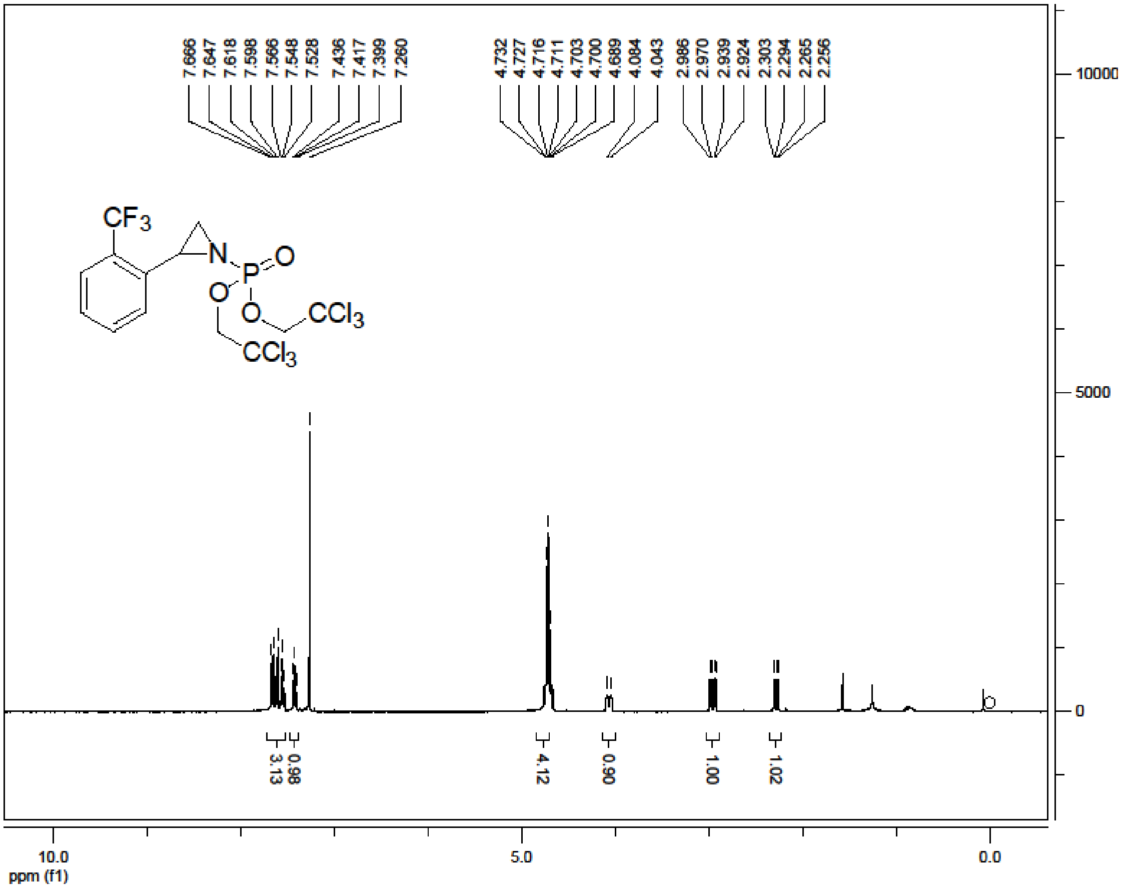

Figure S17. ^{1}H NMR of compound **3e**.

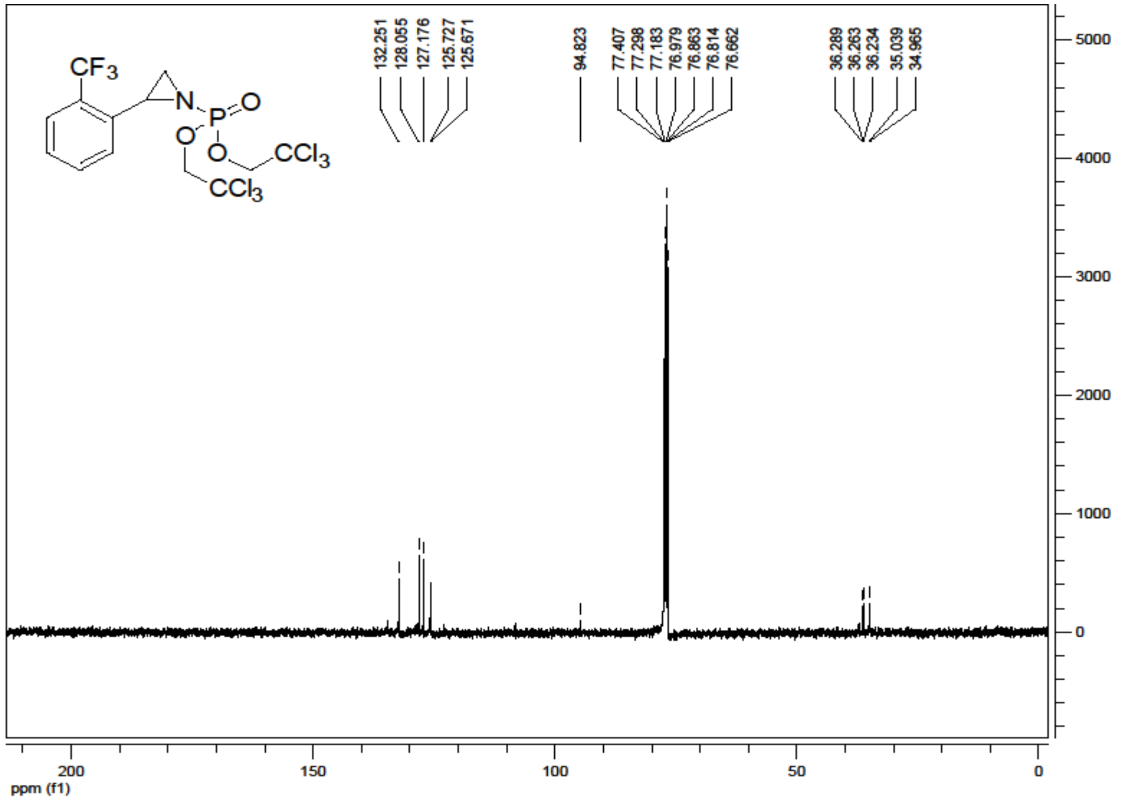

Figure S18. ^{13}C NMR of compound **3e**.

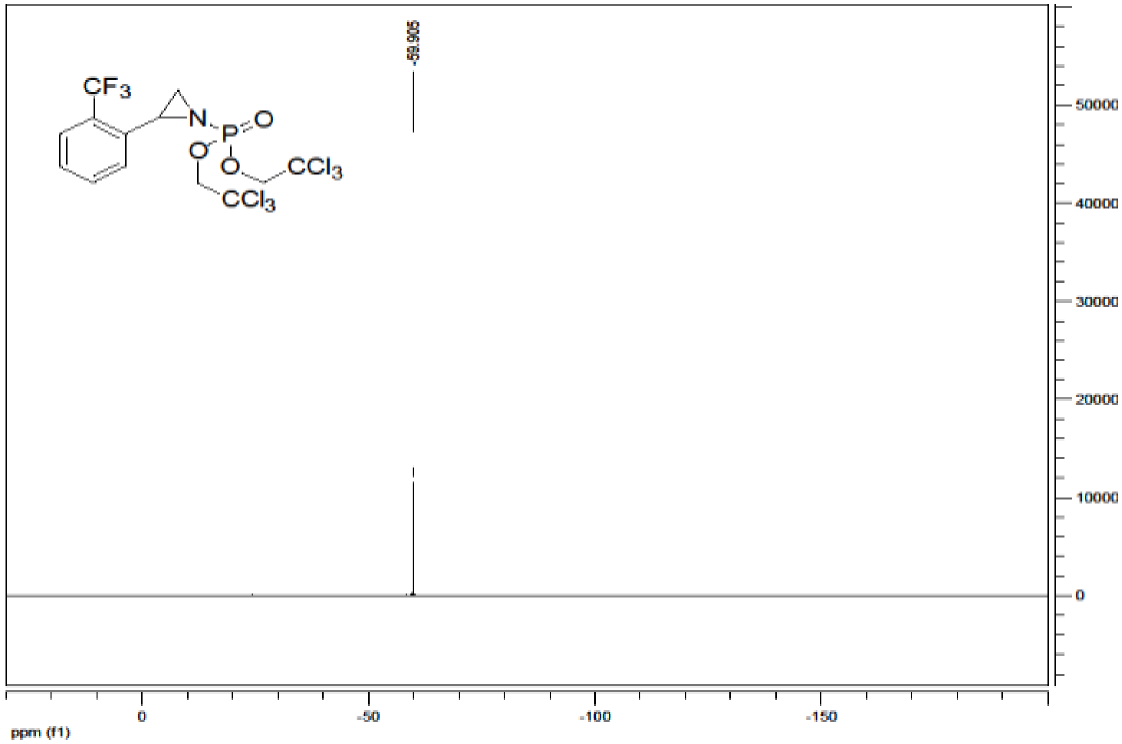

Figure S19. ^{19}F NMR of compound **3e**.

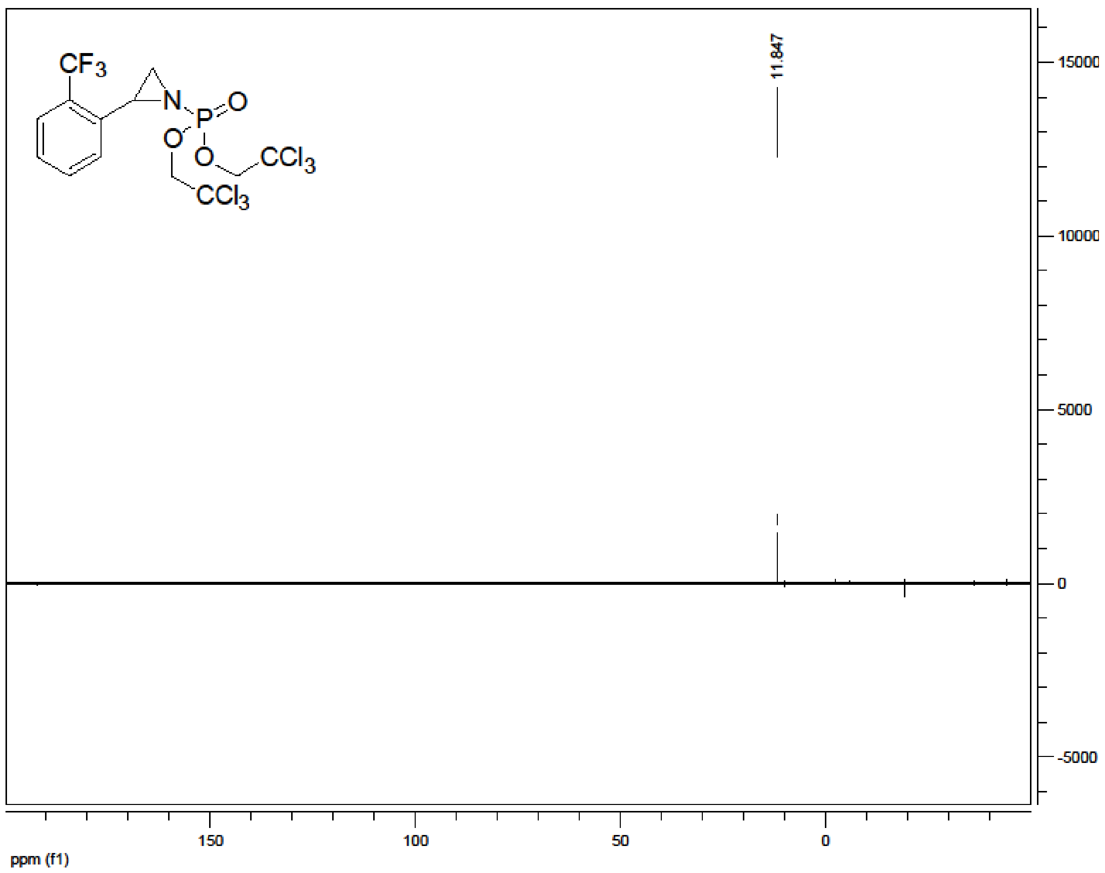

Figure S20. ^{31}P NMR of compound **3e**.

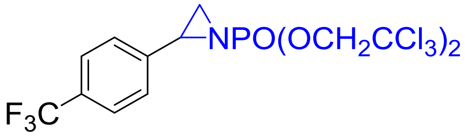

3f

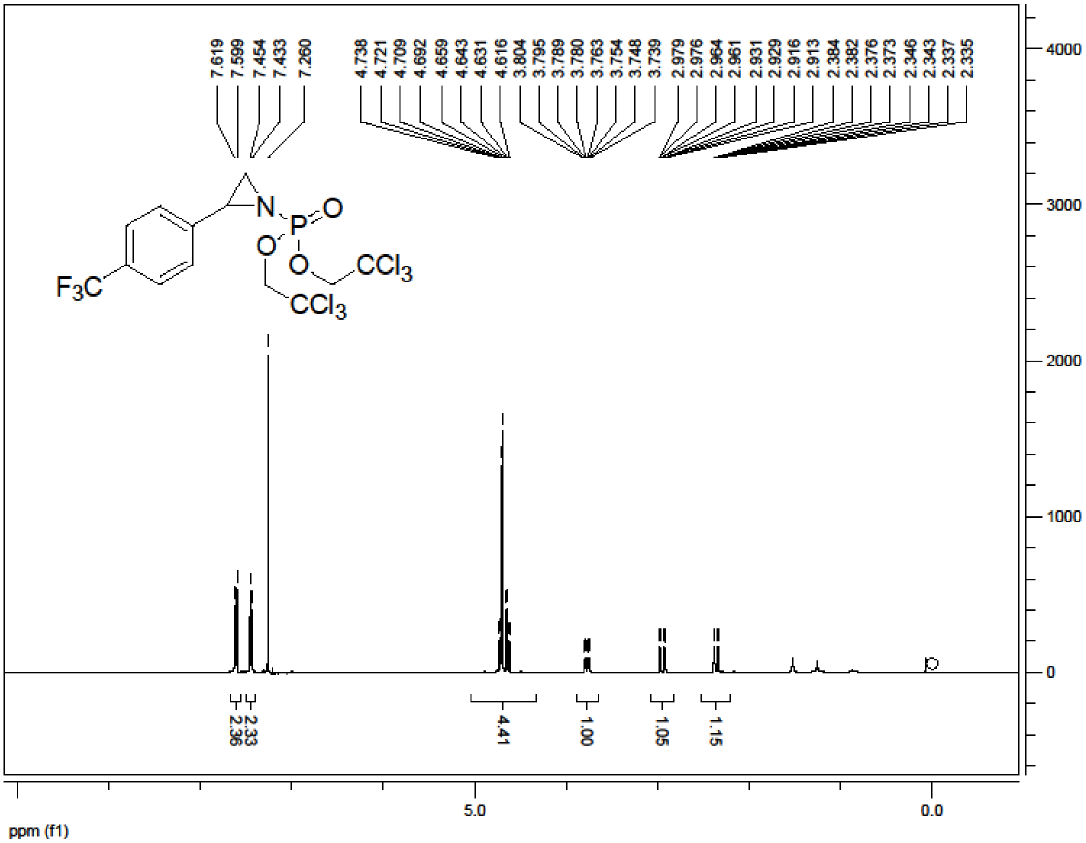

Figure S21. ^{1}H NMR of compound **3f**.

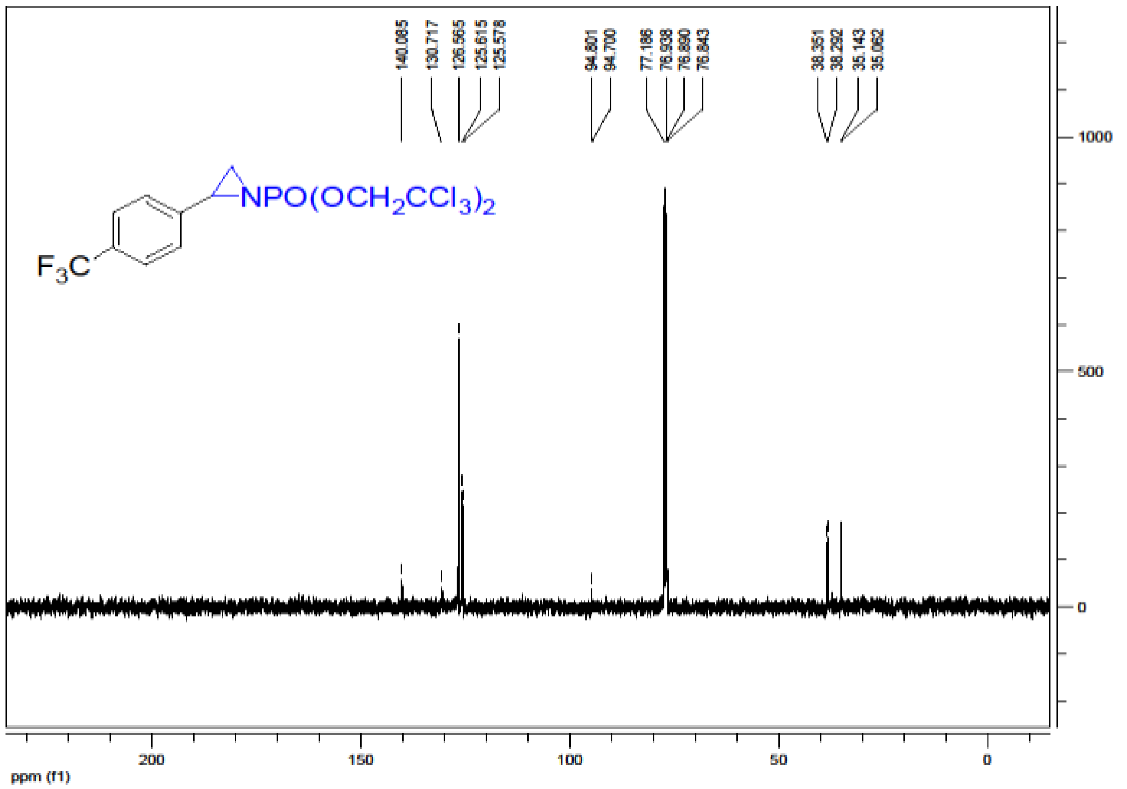

Figure S22. ^{13}C NMR of compound **3f**.

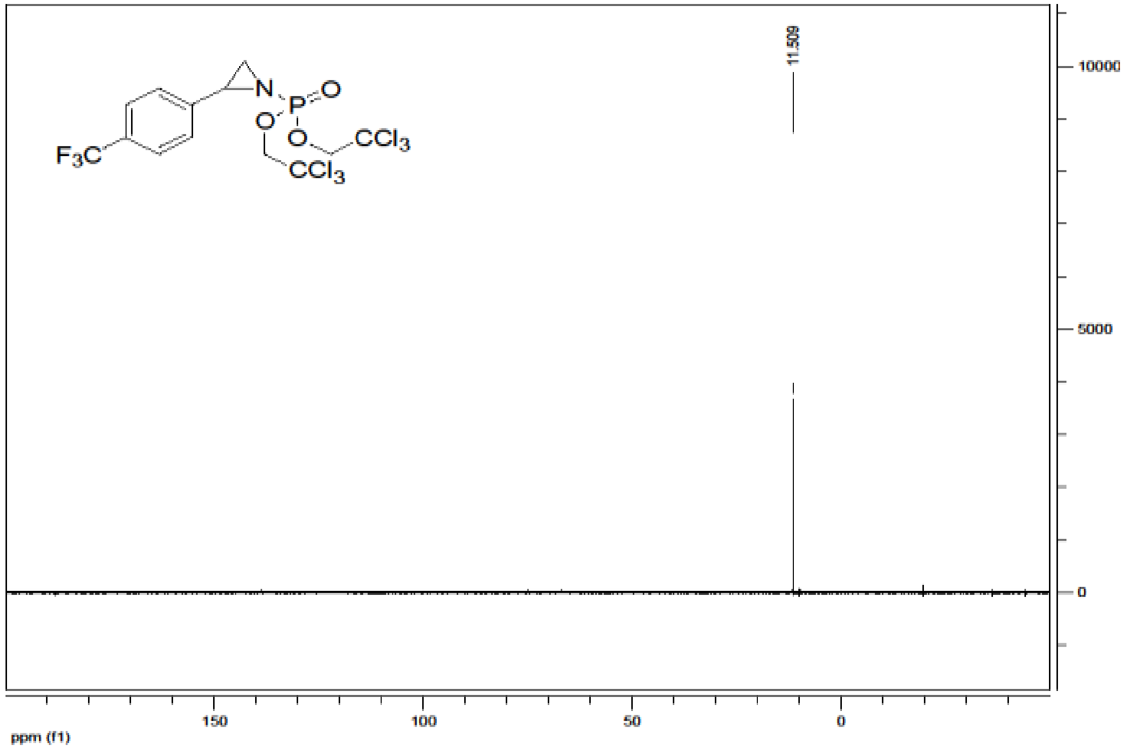

Figure S23. ^{31}P NMR of compound **3f**.

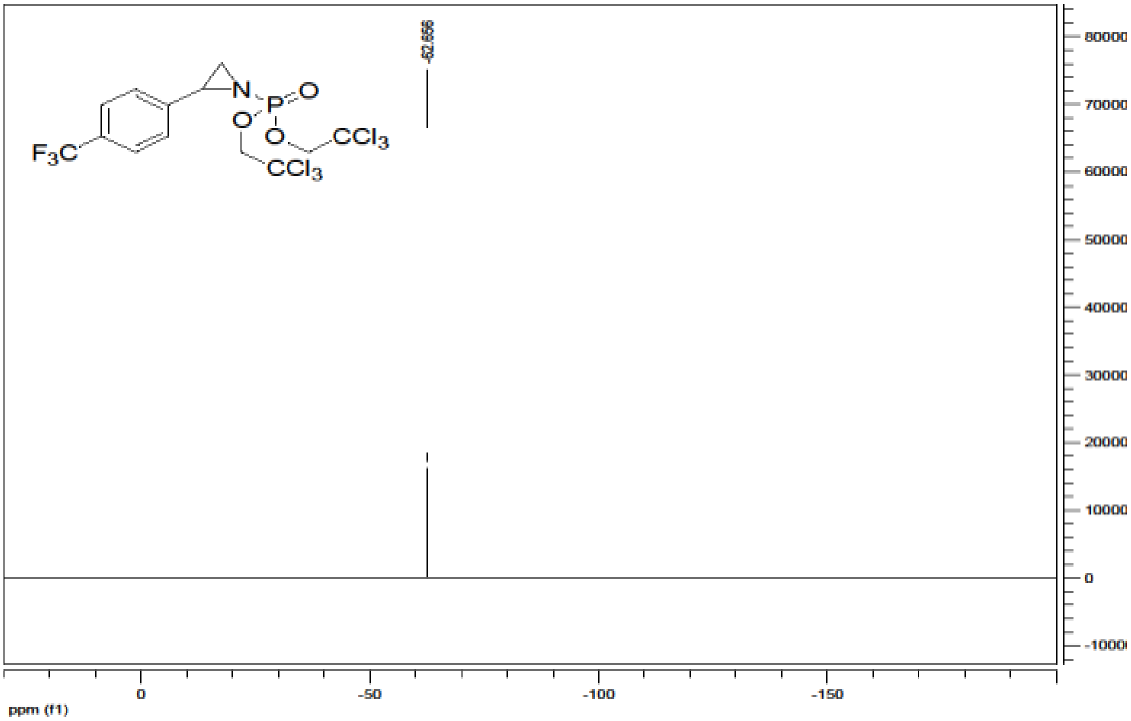

Figure S24. ^{19}F NMR of compound **3f**.

JRT-VI220A-WK2%0.5ML150MIN
C:\EZStart\Projects\Default\Method\JYW-ADH-1%1ml.met
C:\EZStart\Projects\Default\Data\JRT-VI220A-WK2%0.5ML150MIN

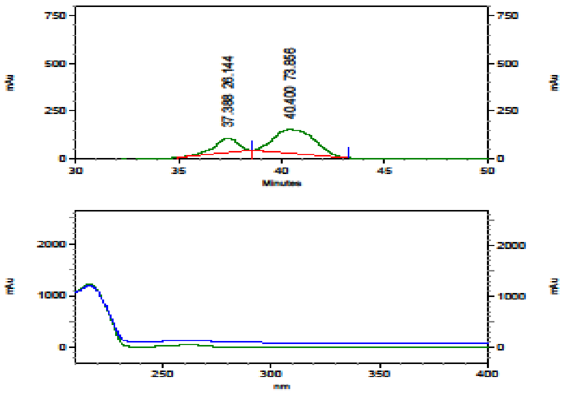

7: 218 nm, 4 nm
Results

Pk #	Name	Retention Time	Area Percent
1		37.388	26.144
2		40.400	73.856
Totals			100.000

Figure S25. HPLC of compound **3f**.

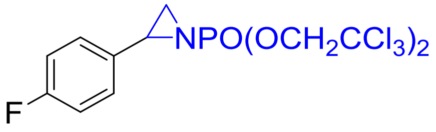

3g

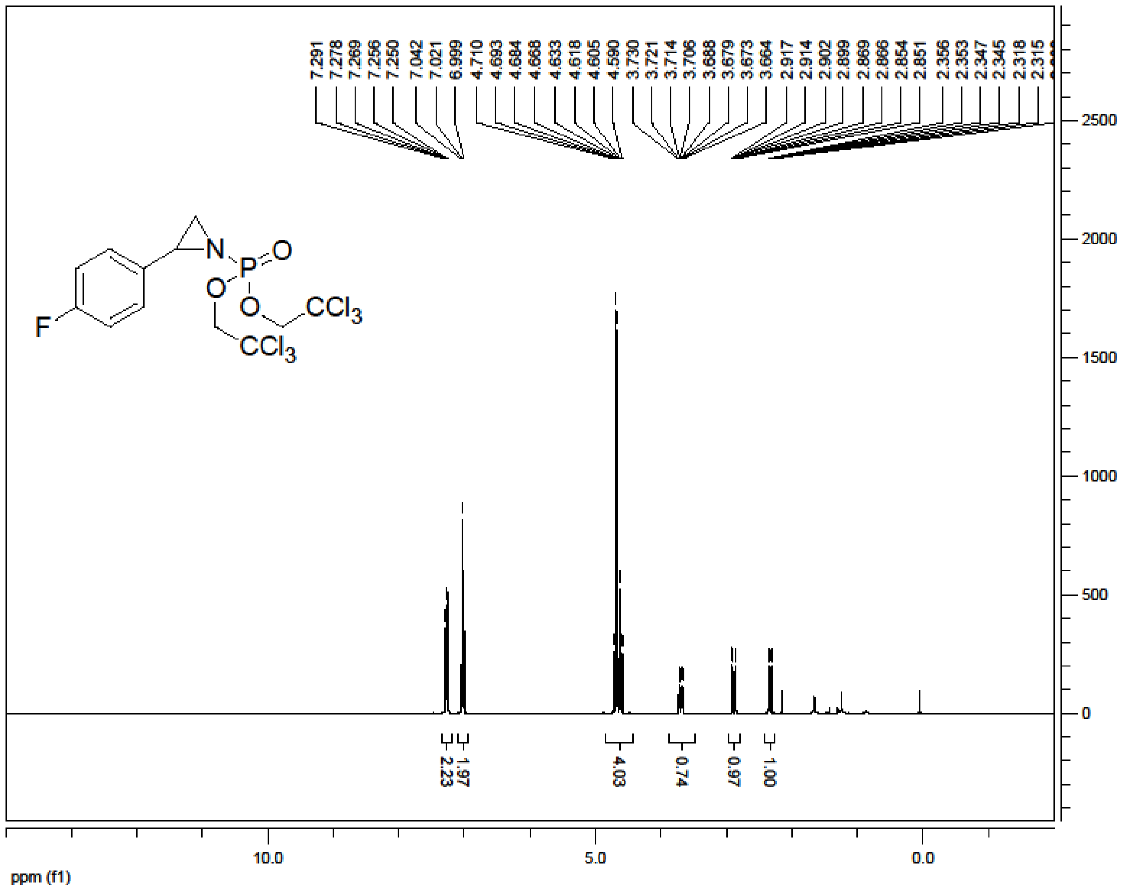

Figure S26. ^{1}H NMR of compound **3g**.

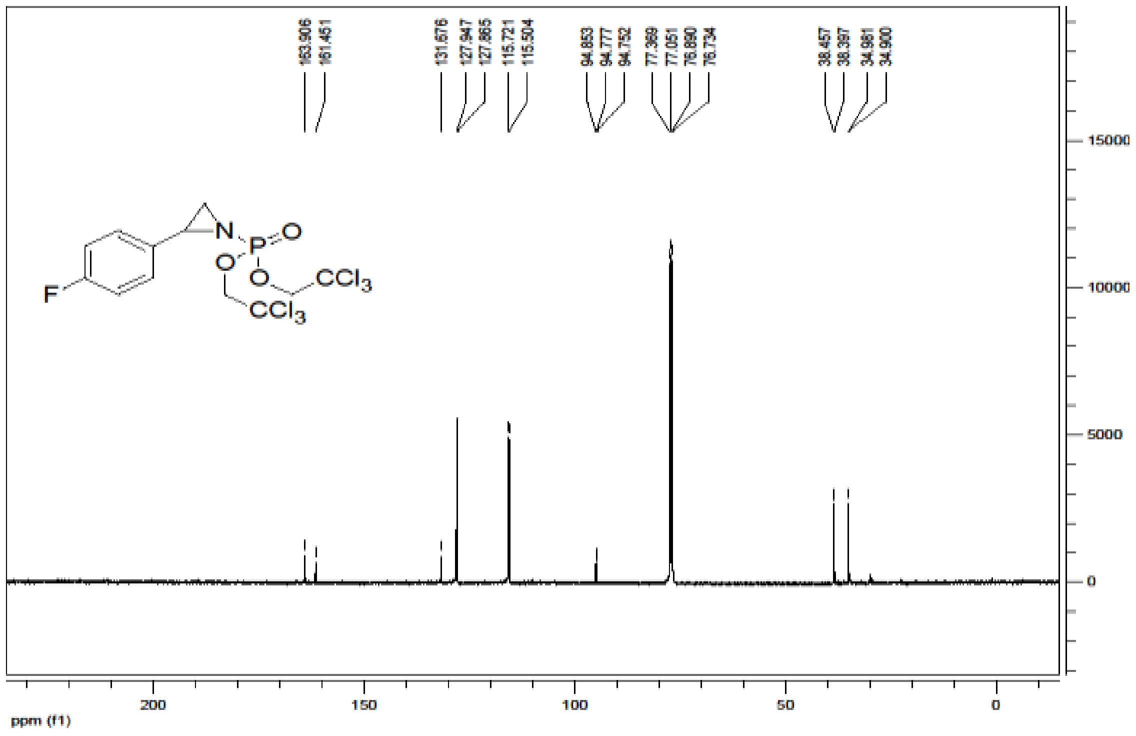

Figure S27. ^{13}C NMR of compound **3g**.

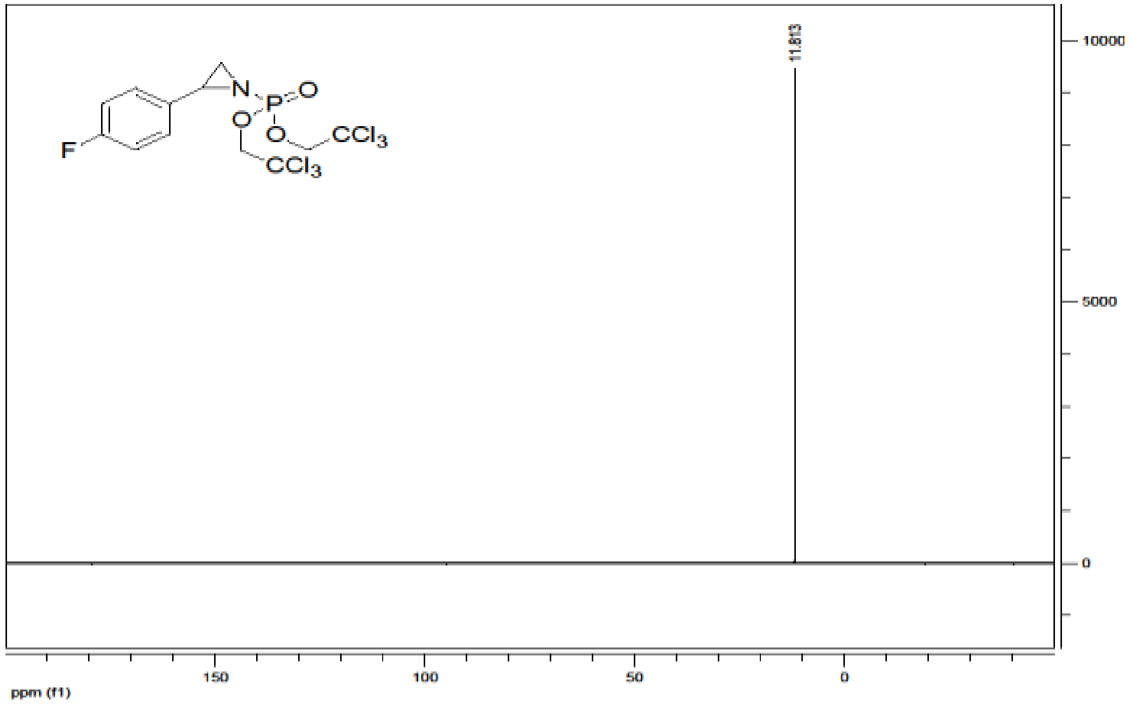

Figure S28. ^{31}P NMR of compound **3g**.

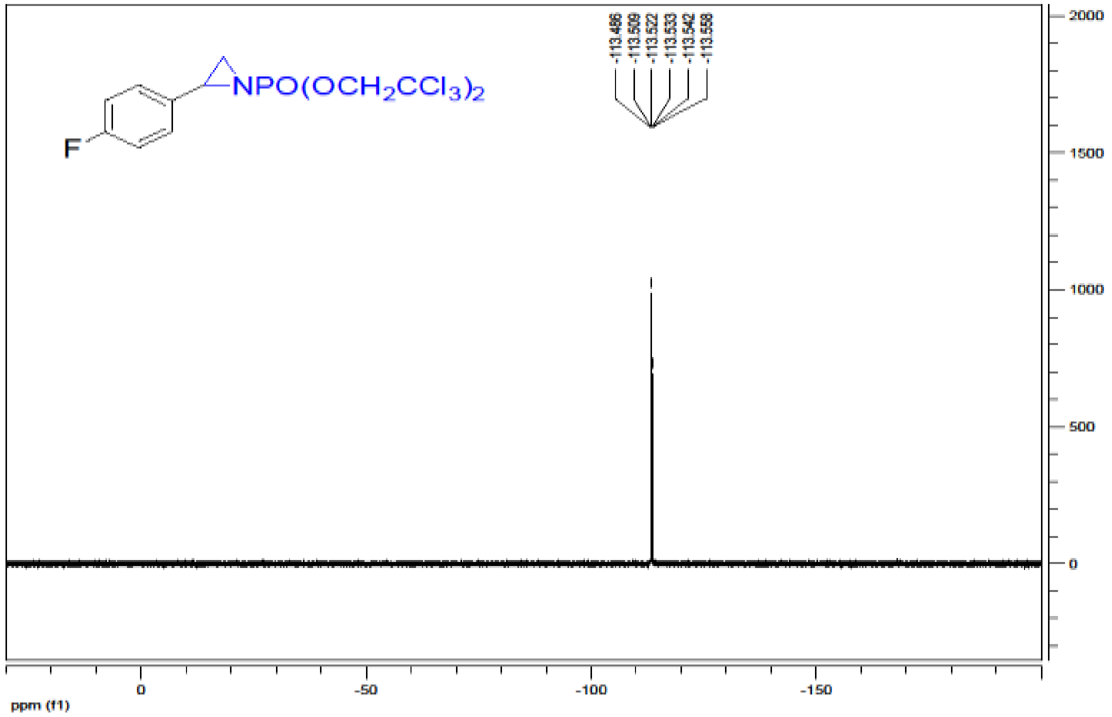

Figure S29. ^{19}F NMR of compound **3g**.

JRT-VI218A-WK2@1ML150MIN
C:\EZStart\Projects\Default\Method\JYW-ADH-1@1ml.met
C:\EZStart\Projects\Default\Data\JRT-VI218A-WK2@1ML150MIN

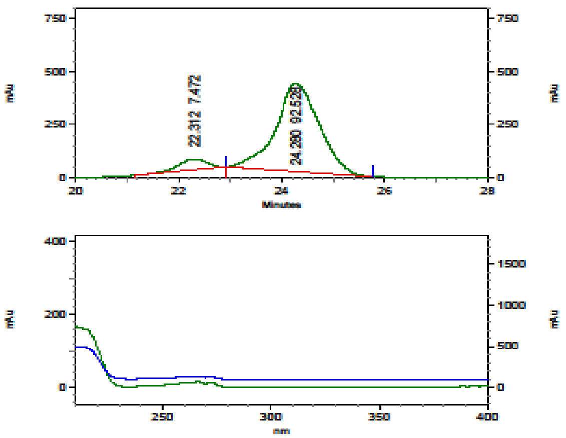

7: 218 nm, 4 nm
Results

Pk #	Name	Retention Time	Area Percent
1		22.312	7.472
2		24.280	92.528
Totals			100.000

Figure S30. HPLC of compound **3g**.

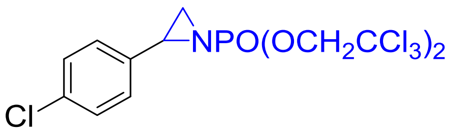

3h

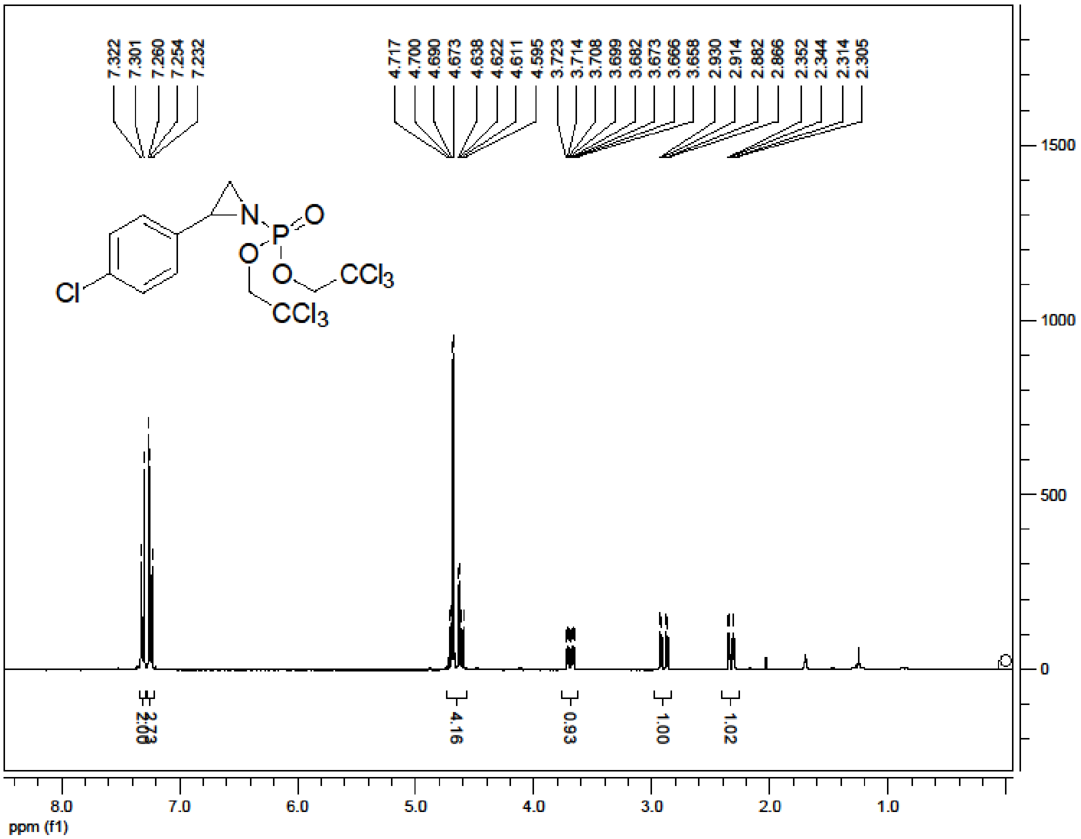

Figure S31. ^{1}H NMR of compound **3h**.

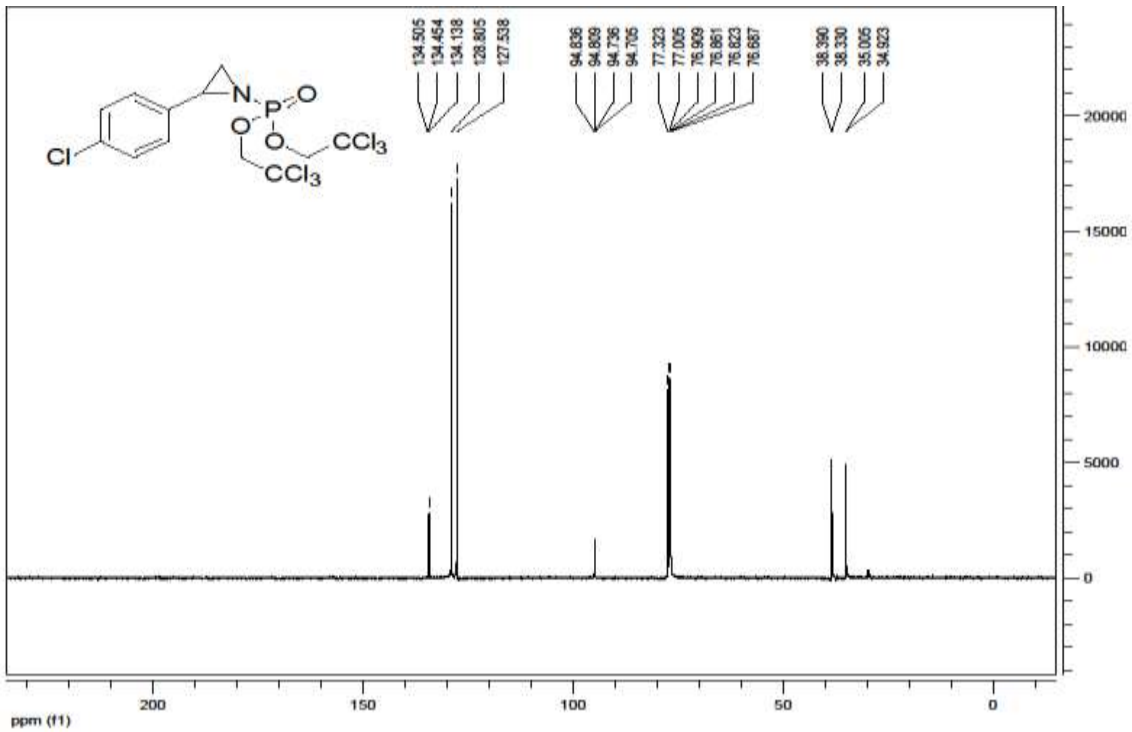

Figure S32. ^{13}C NMR of compound **3h**.

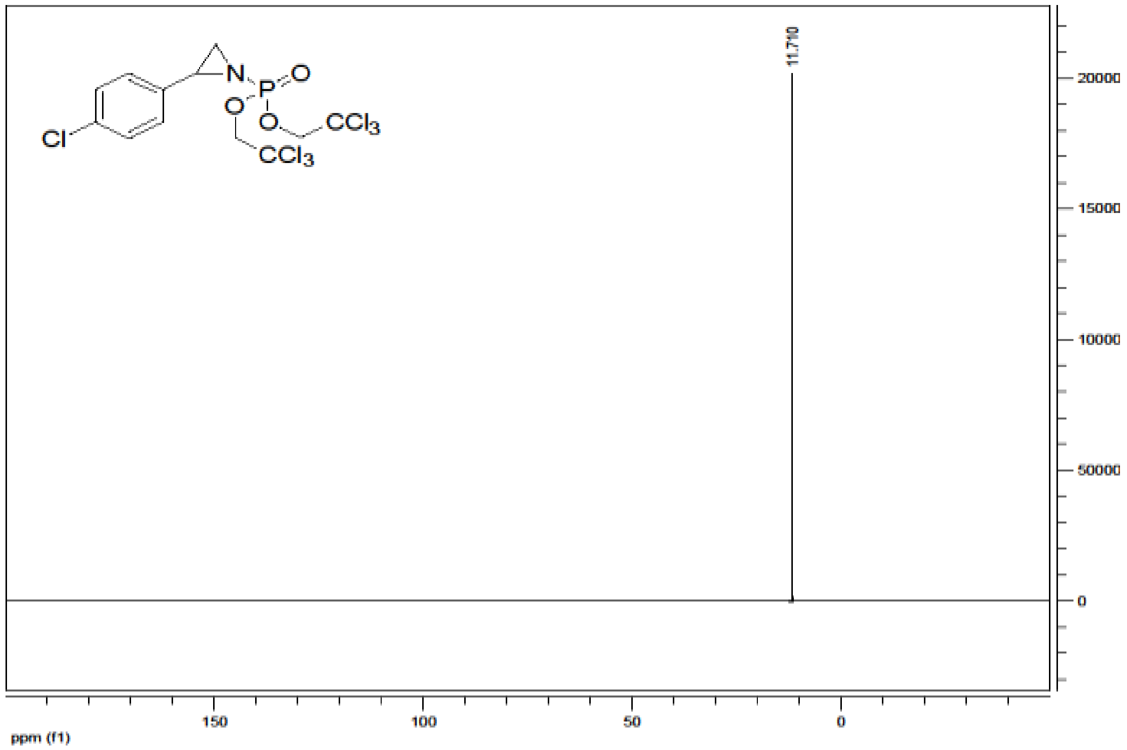

Figure S33. ^{31}P NMR of compound **3h**.

JRT-VI-101WK2%1ML40MIN
C:\EZStart\Projects\Default\Method\shifatest_2,5-dimehoxy.met
C:\EZStart\Projects\Default\Data\JRT-VI-101WK2%1ML40MIN

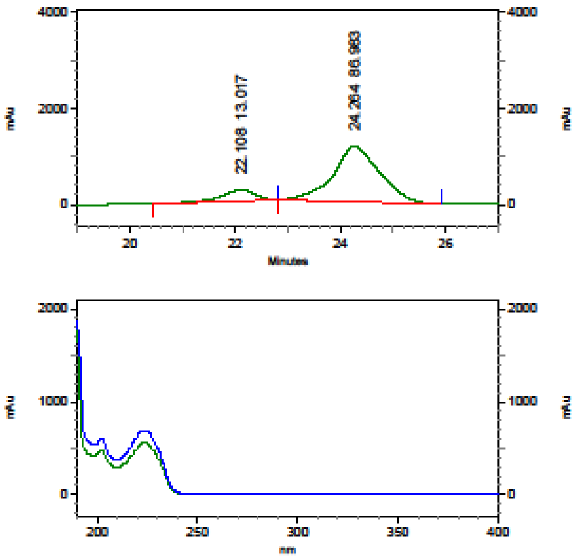

3: 209 nm, 4 nm
Results

Pk #	Name	Retention Time	Area Percent
1		22.108	13.017
2		24.264	86.983
Totals			100.000

Figure S34. HPLC of compound **3h**.

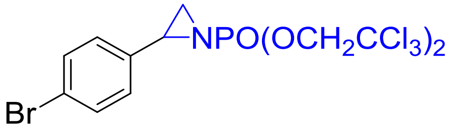

3i

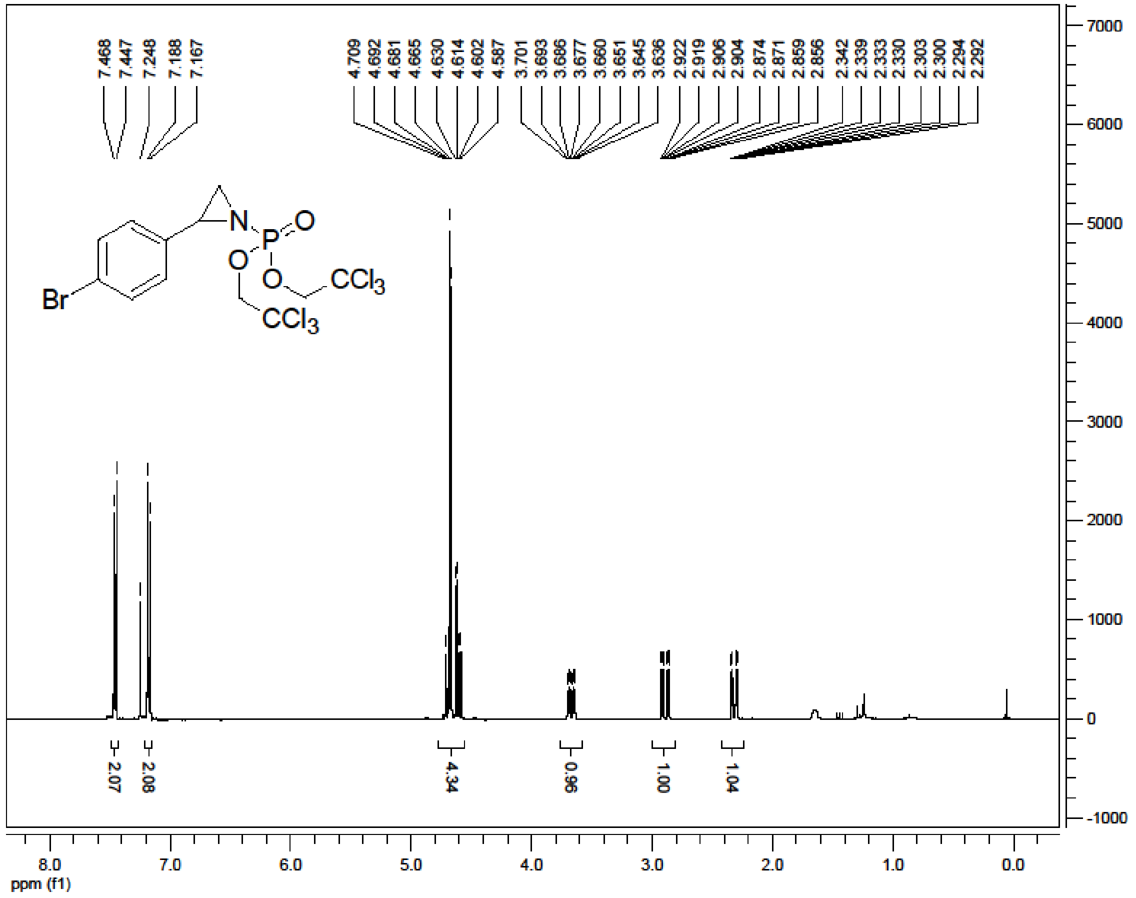

Figure S35. ^{1}H NMR of compound **3i**.

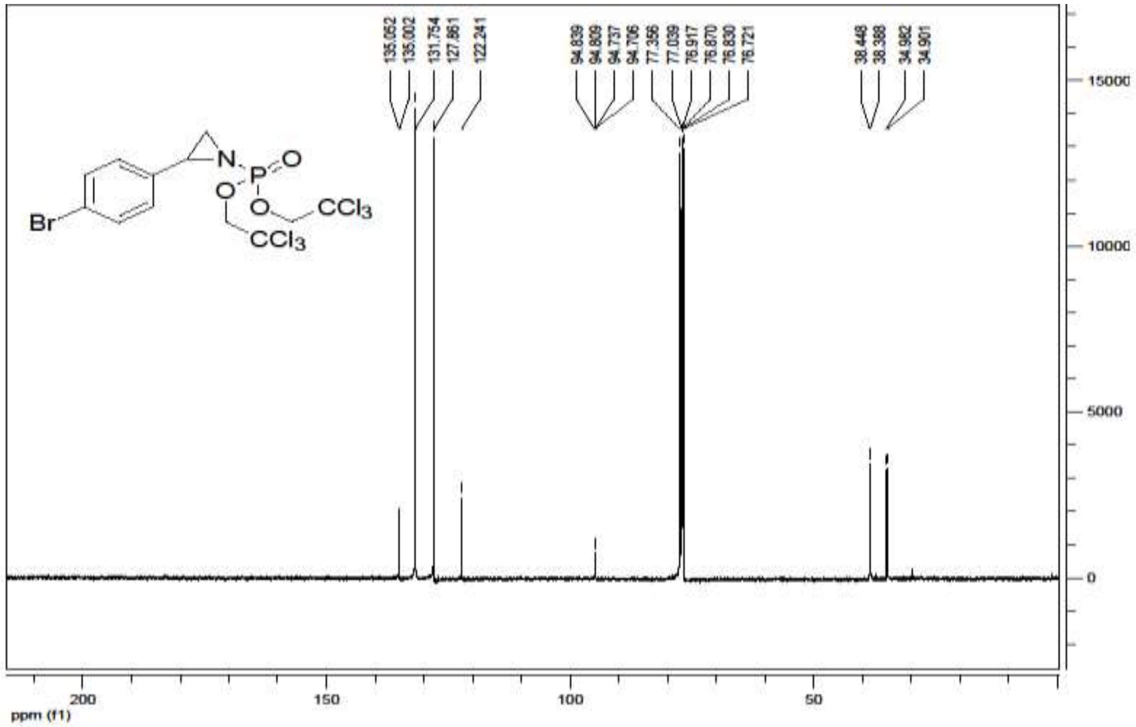

Figure S36. [13] C NMR of compound **3i**.

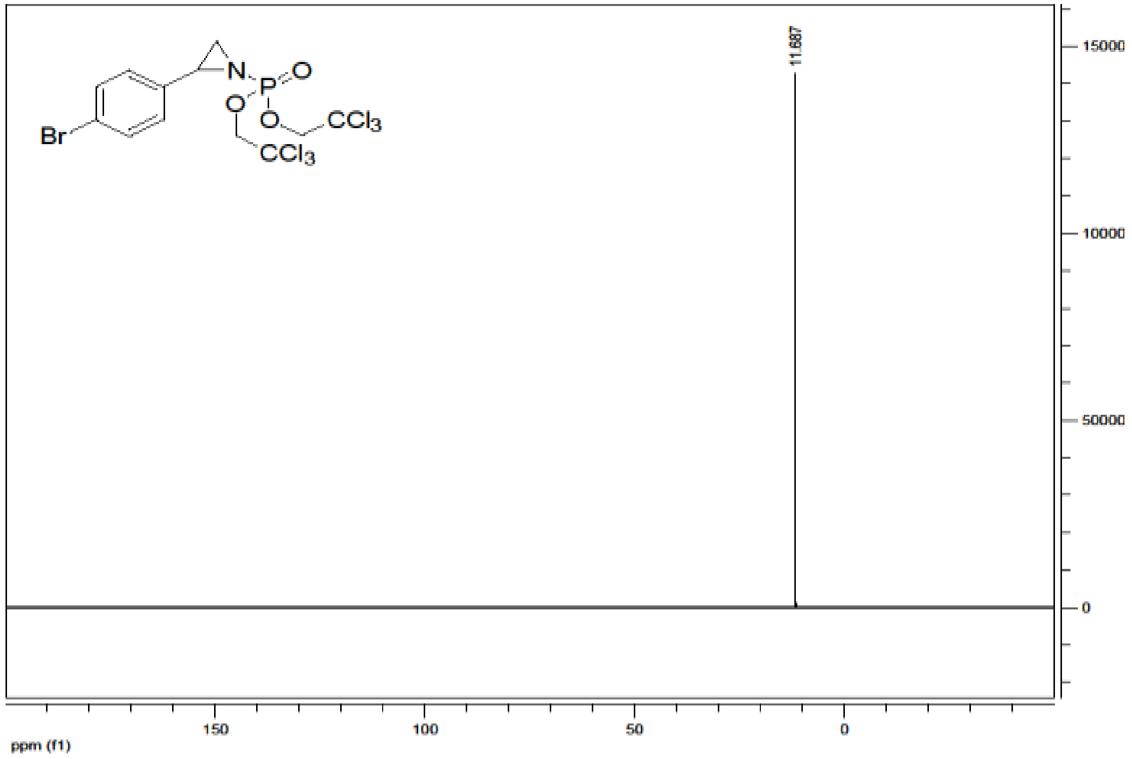

Figure S37. [31] P NMR of compound **3i**.

JRT-VI-218B-WK2%1ML30MIN
C:\EZStart\Projects\Default\Method\shifatest_2,5-dimehoxy.met
C:\EZStart\Projects\Default\Data\JRT-VI-218B-WK2%1ML30MIN

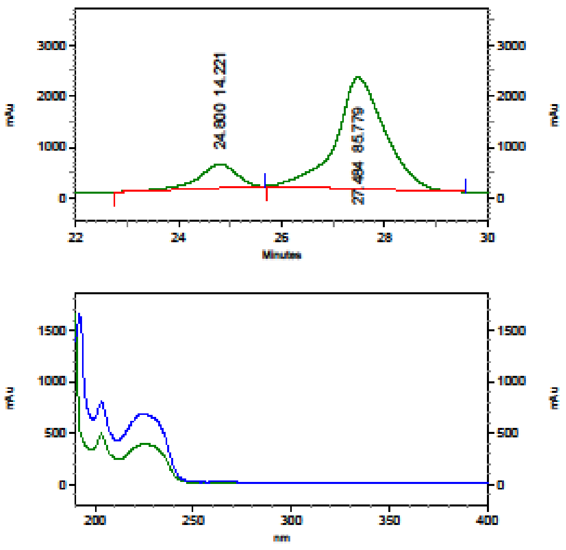

5: 207 nm, 4 nm
Results

Pk #	Name	Retention Time	Area Percent
1		24.800	14.221
2		27.484	85.779
Totals			100.000

Figure S38. HPLC of compound **3i**.

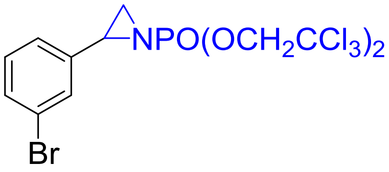

3j

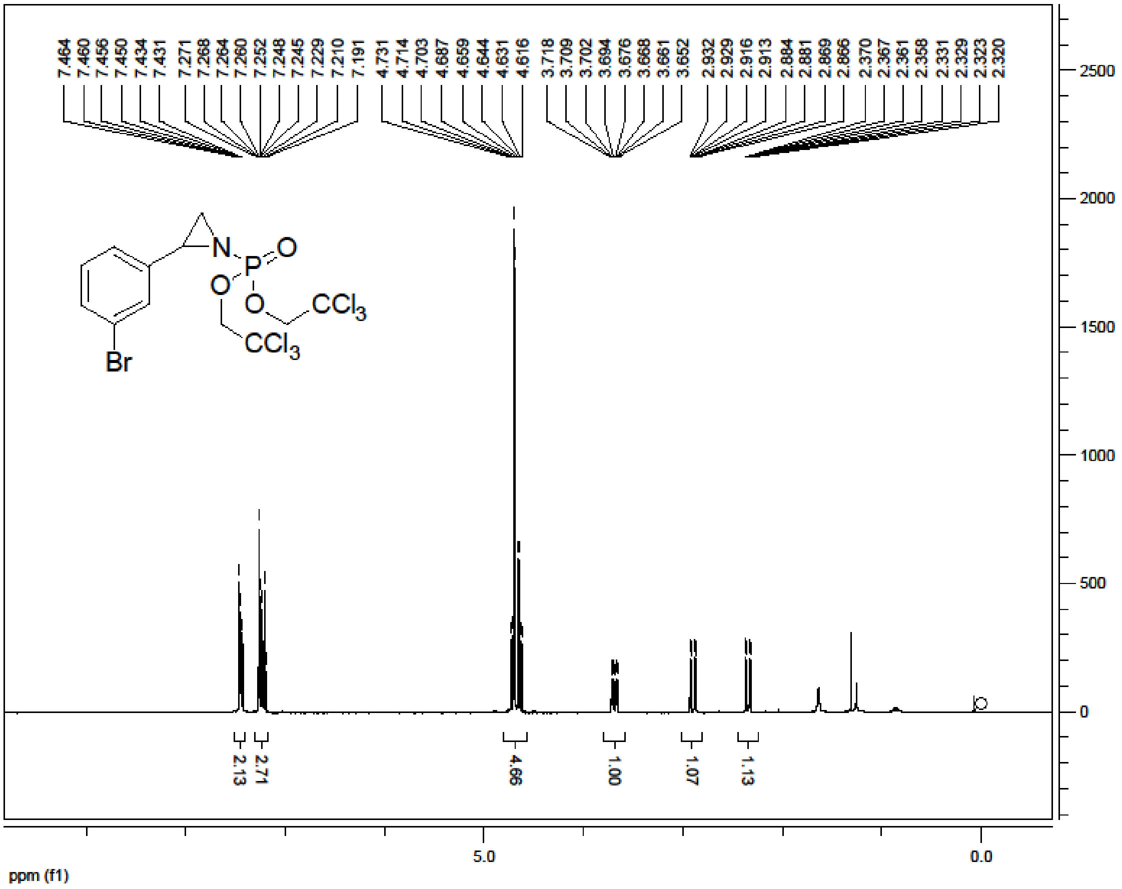

Figure S39. [1] H NMR of compound **3j**.

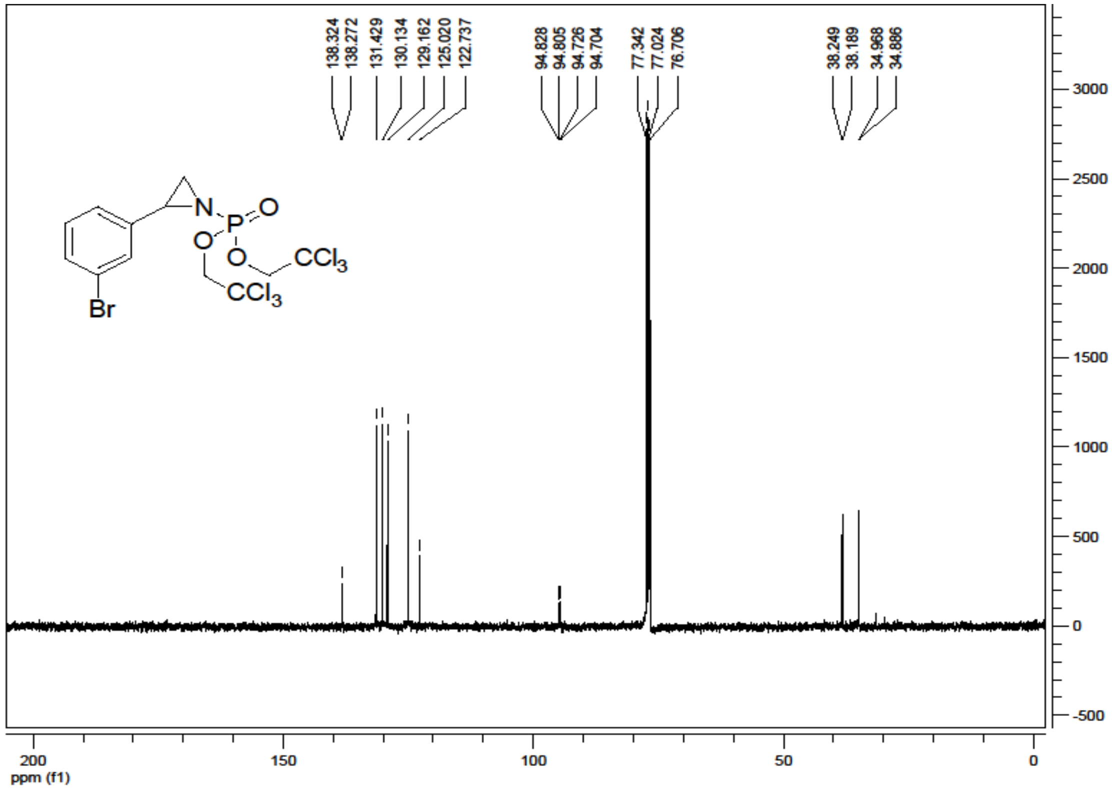

Figure S40. [13] C NMR of compound **3j**.

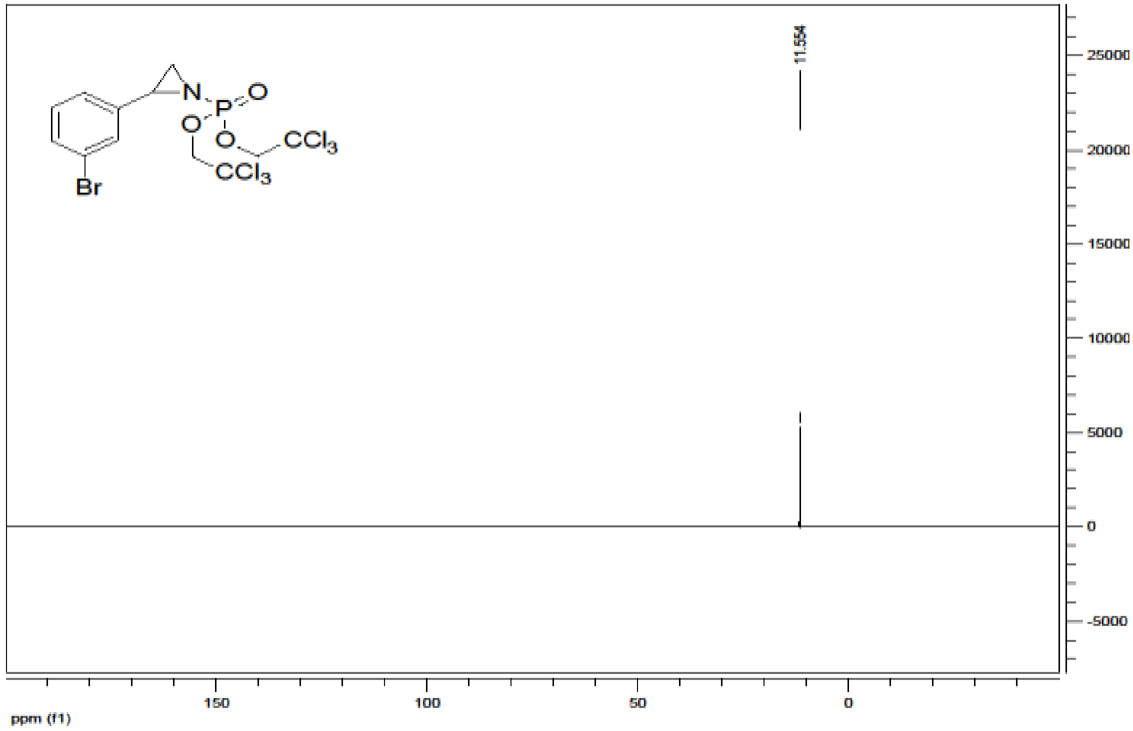

Figure S41. [31] P NMR of compound **3j**.

JRT-VI-111WK2%1ML40MIN
C:\EZStart\Projects\Default\Method\shifatest_2,5-dimehoxy.met
C:\EZStart\Projects\Default\Data\JRT-VI-111WK2%1ML40MIN

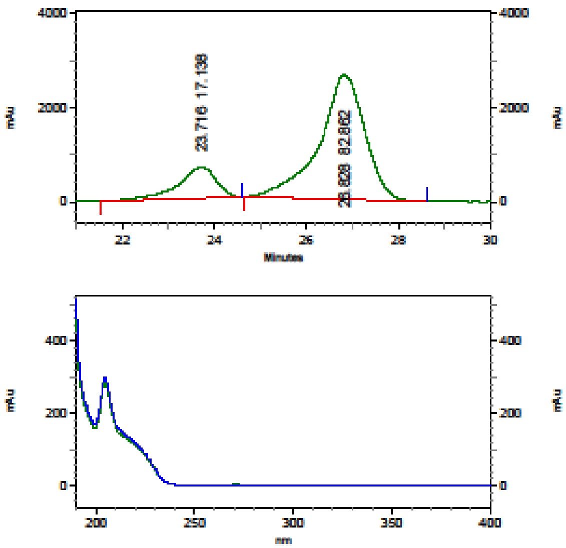

3: 209 nm, 4 nm
Results

Pk #	Name	Retention Time	Area Percent
1		23.716	17.138
2		26.828	82.862
Totals			100.000

Figure S42. HPLC of compound **3j**.

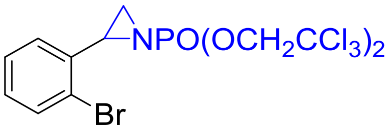

3k

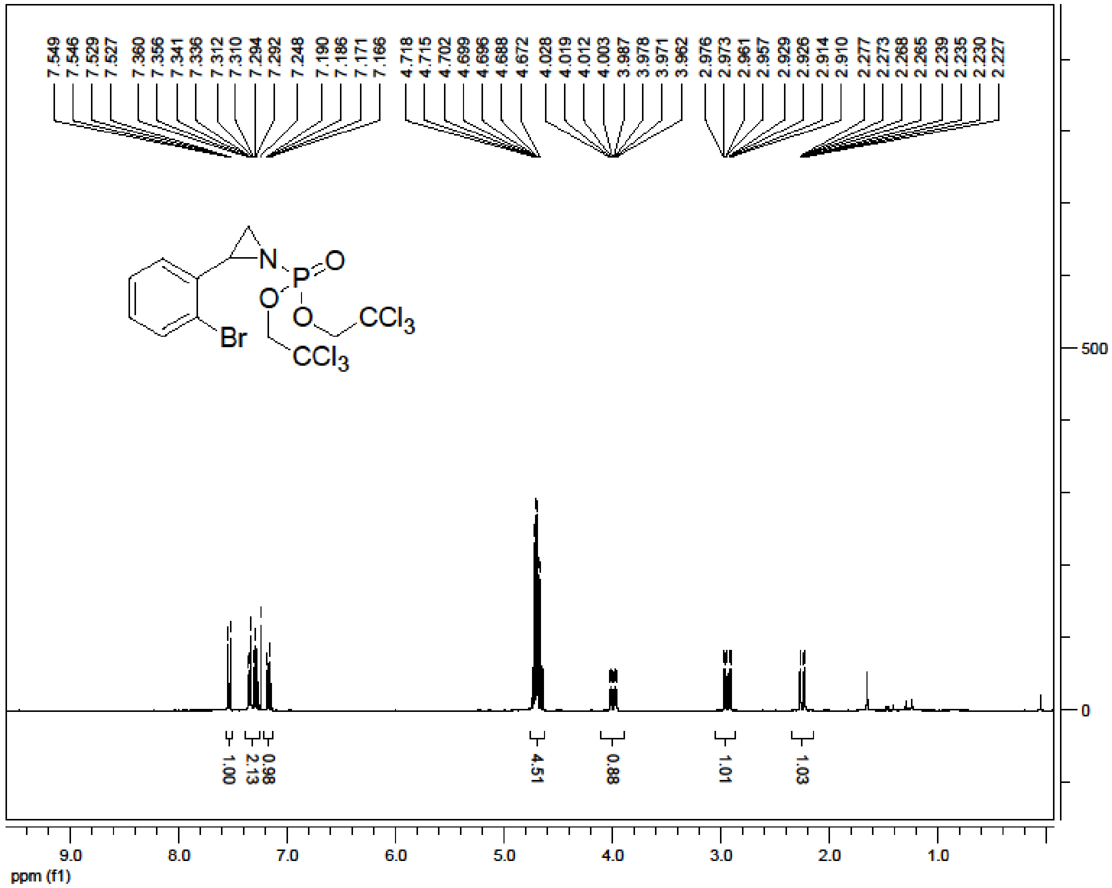

Figure S43. ^{1}H NMR of compound **3k**.

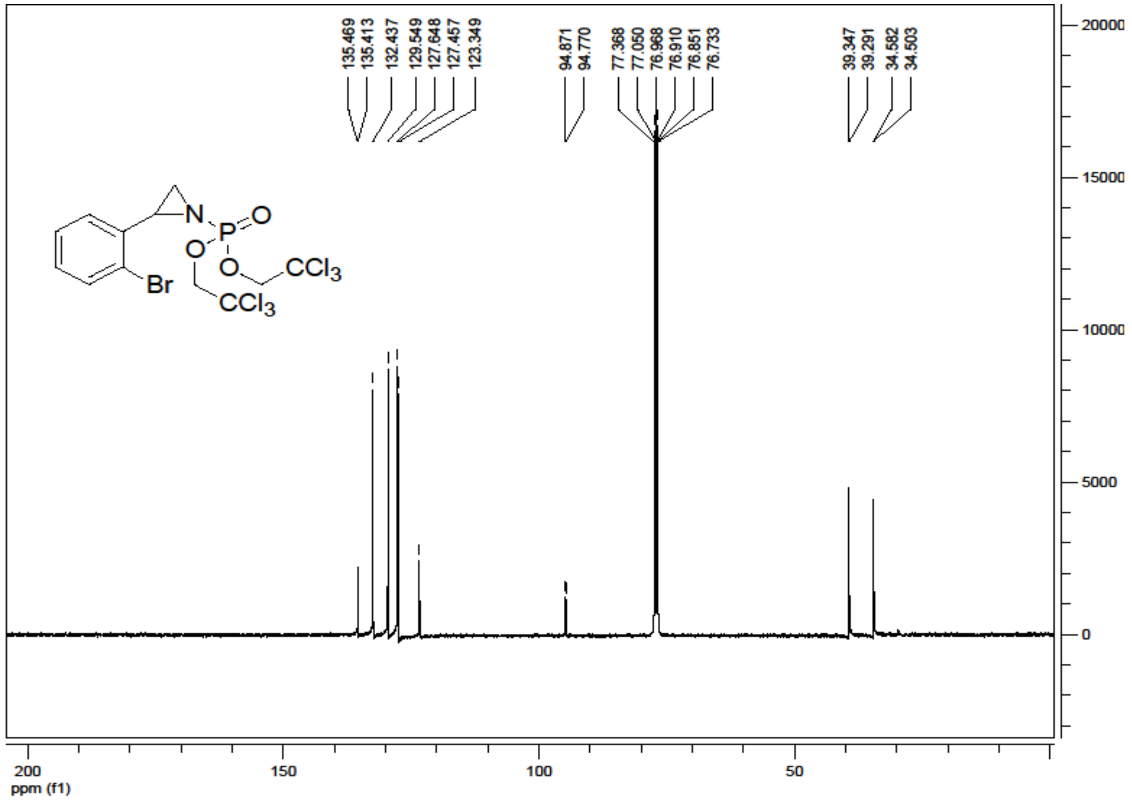

Figure S44. ^{13}C NMR of compound **3k**.

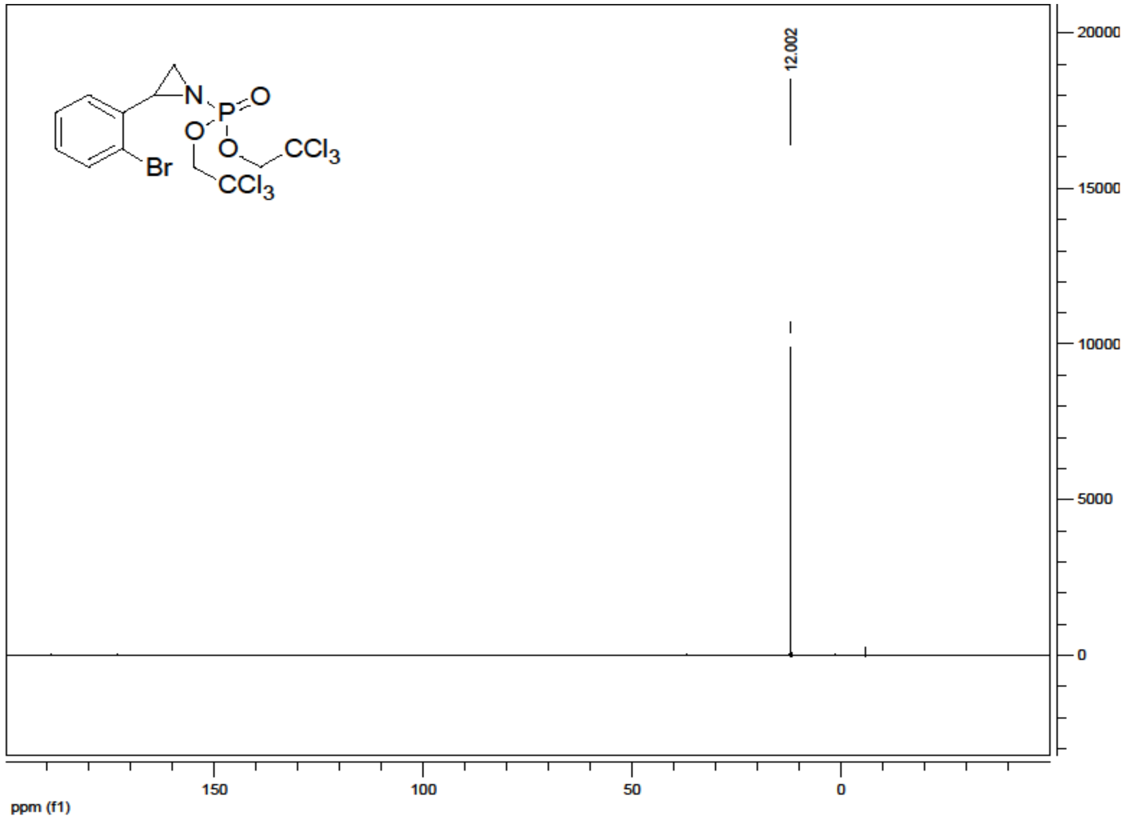

Figure S45. ^{31}P NMR of compound **3k**.

JRT-VI-219B-WK2@1ML150MIN
C:\EZStart\Projects\Default\Method\shifatest_2,5-dimehoxy.met
C:\EZStart\Projects\Default\Data\JRT-VI-219B-WK2@1ML150MIN

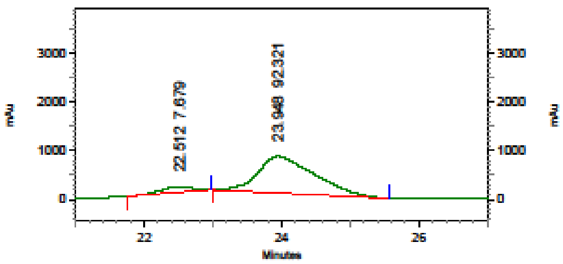

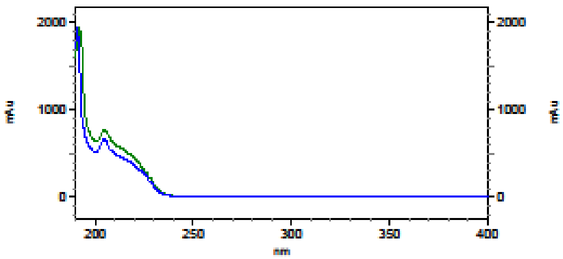

3: 209 nm, 4 nm
Results

Pk #	Name	Retention Time	Area Percent
1		22.512	7.679
2		23.948	92.321
Totals			100.000

Figure S46. HPLC of compound **3k**.
